# Assessment of the biofilm formation capacities of *Staphylococcus aureus* strains Newman and Newman D2C in vitro and in vivo

**DOI:** 10.1038/s41598-025-00521-5

**Published:** 2025-05-08

**Authors:** Ben Wieland, Gubesh Gunaratnam, Linda Pätzold, Noran Abdel Wadood, Georges Pierre Schmartz, Swarnali Kundu, Nikolay Krasimirov Kirilov, Ina Krüger, Mohamed Ibrahem Elhawy, Jacqueline Rehner, Hannah Heintz, Frank Schmitz, Daniela Yildiz, Gabriela Krasteva-Christ, Sören Leif Becker, Karin Jacobs, Markus Bischoff

**Affiliations:** 1 Institute of Medical Microbiology and Hygiene, Saarland University, 66421 Homburg, Germany; 2 Institute of Anatomy and Cell Biology, Saarland University, 66421 Homburg, Germany; 3 Clinical Bioinformatics, Saarland University, 66123 Saarbrücken, Germany; 4 Experimental Physics, Center for Biophysics, Saarland University, 66123 Saarbrücken, Germany; 5 Preclinical Center for Molecular Signaling, Molecular Pharmacology, Saarland University, 66421 Homburg, Germany; 6 Max Planck School Matter to Life, 69120 Heidelberg, Germany

**Keywords:** *Staphylococcus aureus*, Strain Newman, NCTC 8178, Strain Newman D2C, NCTC 10833, Growth kinetics, Adhesion, Biofilm formation, Foreign body-related murine infection model, Microbiology, Bacteriology, Biofilms, Clinical microbiology, Pathogens

## Abstract

*Staphylococcus aureus* is a major cause of implant-associated infections (IAIs). The ability of this Gram-positive bacterium to cause IAIs is closely related to its capacity to attach to and to form biofilms on the implant material. Biofilm formation of *S. aureus* on artificial surfaces is usually mimicked in the laboratory by simple microplate-based in vitro assays and often involves type culture collection preserved laboratory strains such as SA113 (ATCC 35556), Newman (NCTC 8178), and Newman D2C (NCTC 10833, ATCC 25904). The latter two strains are phylogenetically closely related and often inadvertently indicated as strain “Newman” in publications, albeit of the fact that strain Newman D2C harbors among others mutations in the global regulatory loci *agr* and *sae*, which strongly impact the phenotypic behavior of this strain. Wondering how the genetic differences between strains Newman and Newman D2C alter the biofilm formation capacities of these two strains in vitro and in vivo, we tested here the adhesion behavior and biofilm formation capacities of both strains on different kinds of artificial surfaces (tissue culture-treated bottoms of 96-well polystyrene microplates and polyurethane-based peripheral venous catheter [PVC] tubing). Additionally, we determined their ability to cause infection in a foreign body-related murine infection model. Our studies revealed that the Newman and Newman D2C derivatives kept at Saarland University, Germany, differ significantly in their abilities to attach to microplate well bottoms and PVC tubing, and to form biofilms in various static and dynamic in vitro assays. However, when the biofilm formation capacities of both strains were determined in an in vivo infection model, rather comparable bacterial loads were observed. These findings suggest that biofilm formation capacities of *S. aureus* strains may differ substantially in vitro and in vivo. Additionally, researchers working with strains Newman and Newman D2C should be aware that both strains differ substantially in their phenotypic behavior, and that both strains should be indicated correctly to allow for a better comparison of data obtained with these strains in different laboratories.

**Supplementary Information:**

The online version contains supplementary material available at 10.1038/s41598-025-00521-5.

## Introduction

The Gram-positive bacterium *Staphylococcus aureus* is a major global pathogen and a common cause of implant-associated infection (IAIs), including orthopedic devices, intravascular stents, prosthetic valves, and different kinds of vascular catheters^1–5^. Its epidemiological success in causing IAIs can be largely attributed to its ability to adhere to abiotic surfaces and to form biofilms on the implant material, which are bacterial aggregates embedded in a matrix of extracellular polymeric substances that usually tightly adhere to the abiotic surface^6^. The biofilm formation process can be divided into several steps, starting with the initial adhesion of the bacterium to the implant material, which is considered a crucial step in initial biofilm formation^7^. Depending on the physicochemical properties of the implant surface (topography, hydrophobicity, and electrostatic characteristics), the bacterial cell may attach stronger or more loosely to the artificial surface^7–11^. *S. aureus* manages to attach more firmly to hydrophobic surfaces, which allow a denser interaction between a larger number of bacterial cell macromolecules and the substratum. In comparison, the adhesion of this bacterium to hydrophilic surfaces seems to be dominated by a smaller number of bacterial cell adhesin-substratum interactions^7^. Nanostructured surfaces were also reported to decrease the initial adhesion capacity of *S. aureus* to the artificial surfaces^9^, while surface asperity in the micrometer range may promote the adhesion by increasing the contact area^12^. Another important factor affecting the initial bacterial adhesion to implanted medical devices is bodily fluids, as implanted medical devices are decorated within seconds by host factors present in the bodily fluid(s) the medical device is in contact with^13^. We recently demonstrated that a decoration of catheter tubing with human blood plasma significantly decreased the initial adhesion capacity of *S. aureus* to the implant^14^, while the same treatment might have an enhancing effect on the adhesion capacities of other biofilm-forming microbial pathogens, such as *Candida albicans*^15^. The attachment of the bacterial cell to the abiotic surface is governed in *S. aureus* by proteinaceous and non-proteinaceous cell wall macromolecules^16^. A large number of cell wall-anchored and surface-associated proteins produced by *S. aureus* have been demonstrated to contribute to the attachment of the bacterial cell to the artificial surface and to form a biofilm matrix, including β-hemolysin (Hlb), collagen-binding adhesin (Cna), clumping factor B (ClfB), extracellular adherence protein (Eap), fibronectin binding proteins A and B (FnbA and FnbB), protein A (Spa), and *S. aureus* surface protein G (SasG) (reviewed in^17^). Another important group of cell wall macromolecules contributing to the adhesion of *S. aureus* to abiotic surfaces are teichoic acids, which affect, amongst others, the net charge of the cell wall and its hydrophobicity^16,18,19^. The stable bacterial adhesion to the implant material is followed by the formation of a microcolony (i.e., cell aggregation and extracellular matrix production), and biofilm maturation, the remodeling of the biofilm architecture by bacterial secretion factors (reviewed in^6,17^). The extracellular matrix can be seen as a kind of glue encasing the whole bacterial cell population, which is usually composed of host factors, secreted and cell lysis-released proteins, polysaccharides, and extracellular DNA (eDNA). However, the composition of the extracellular matrix formed by *S. aureus* may differ substantially, depending on the strain background and/or the environmental conditions the bacterial cells are exposed to^20^. A major constituent of the extracellular matrix produced by many *S. aureus* strains reported to produce thick biofilms under in vitro conditions is polysaccharide intercellular adhesin (PIA), an exopolymer composed of β-1,6-linked *N*-acetylglucosamine, which is synthesized by enzymes encoded by the *ica* locus^21^. Expression of the *ica* locus in *S. aureus* is tightly controlled by a multitude of transcriptional regulatory factors and is strongly affected by environmental stimuli such as anaerobiosis, glucose, iron availability, or high salt concentrations^20^. Regulatory loci of particular interest for PIA production are *agr* (accessory gene regulator), *sae* (*S. aureus* exoprotein expression), and *sarA* (staphylococcal accessory regulator A), which are in part interconnected. Earlier work demonstrated that the expression of *agr* reduces the capacity of *S. aureus* to form biofilms^22^. Several proteins/peptides whose expression are promoted by *agr*, such as nucleases (encoded by *nuc1* and *nuc2*) and phenol soluble modulins (encoded by *psma* and *psmß*) are also important for the remodeling of the biofilm architecture during the maturation phase and for biofilm dispersal, in which extracellular matrix-embedded bacterial cells are released in concert with proteases to allow the bacterium to return to a planktonic lifestyle^17^. A major negative impact of *agr* on biofilm formation of *S. aureus* is also suggested by the fact that many *S. aureus* isolates forming a strong PIA-dependent biofilm under in vitro conditions display low to no *agr* expression^23,24^, and that the strong PIA-dependent biofilm-producing strain SA113^25^ (ATCC 35556) is a natural *agr* mutant. The impact of certain genes or substances on the biofilm formation of *S. aureus* under in vitro conditions is often studied in a technically simple 96-well microplate-based biofilm assay^26^, which can be conducted under either static or dynamic conditions and often involves *S. aureus* strains such as SA113, UAMS-1^27^ (ATCC 49230), Newman D2C^28^ (NCTC 10833, ATCC 25904), and Newman^29^ (NCTC 8178). The latter two strains are phylogenetically closely related and probably originate from a coagulase-producing strain isolated from a case of osteomyelitis^30^. The relatedness of both strains is also reflected by the fact that both strains share a very rare *saeS* allele leading to a proline at amino acid position 18 of the sensor kinase SaeS of the SaePQRS regulatory system, resulting in an over-active SaeRS two-component system^31^. Strains Newman and Newman D2C are also often inadvertently reported as the other strain in publications (i.e., Newman as ATCC 25904 and Newman D2C as Newman). In fact, screening the ATCC reference library for strain ATCC 25904 revealed that many of the listed publications refer to or abbreviate strain ATCC 25904 as strain Newman (12/24 listed publications referring to ATCC 25904 published in 2023 and later^32–43^. However, this probably unintentional mislabeling of Newman D2C as Newman is highly problematic, as both strains differ substantially in their phenotypic behavior in vitro and in vivo^44–46^. Strains Newman and Newman D2C also differ in their capacities to form biofilms in 96-well microtiter-based static in vitro assays, with the latter strain being reported to form a moderate biofilm in this type of assay that is less dependent on PIA production than SA113^47^, while the former strain is mostly considered a rather poor biofilm former under these test conditions^21,48,49^. However, reports on the biofilm formation capacity of strain Newman in this assay type are conflicting, ranging from no clear biofilm formation to biofilm formation capacities rather comparable to those seen with strain Newman D2C in this assay^21,30,46,48–50^. Motivated by the work of Sause and colleagues^44^ and the discrepancies reported for the biofilm formation capacities of strain “Newman” in the literature, we directly compared here the in vitro growth behavior of strains Newman and Newman D2C in batch culture, their initial adhesion on artificial surfaces, their biofilm formation properties in different in vitro assays, and their capacities to form a biofilm in vivo.

## Results and discussion

### Genomic composition of the *S. aureus* Newman and Newman D2C derivatives used at Saarland University

**Fig. 1:**
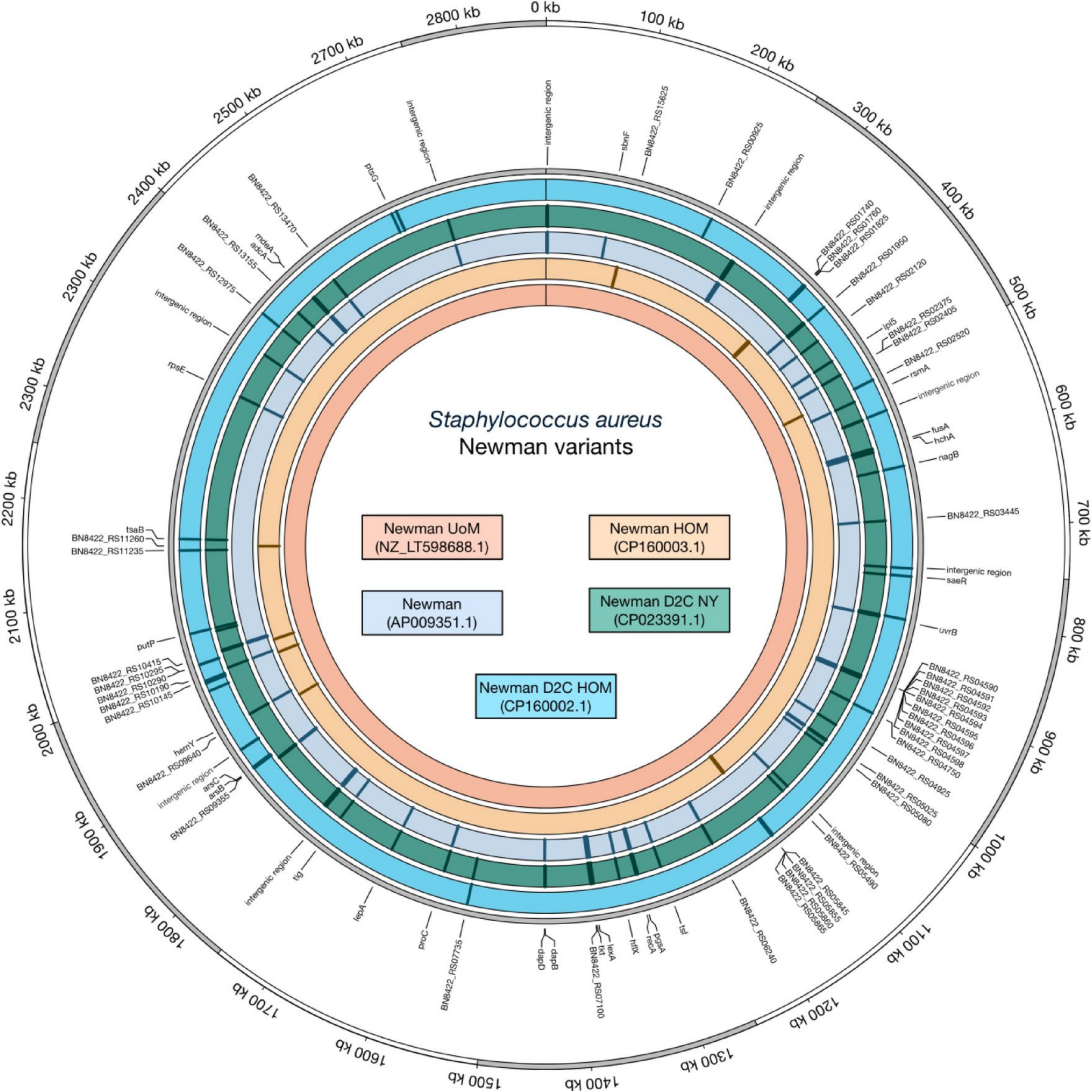
Whole-genome sequencing reveals disparities between the Newman/Newman D2C derivatives in stock at the Institute for Medical Microbiology and Hygiene (Homburg, Germany) and published Newman/Newman D2C reference genomes. A visualization of *S. aureus* Newman/Newman D2C genetic diversity is shown on a circular map of the chromosome of Newman UoM (LT598688.1). The first ring from the outside shows the scale of the chromosome in kilobases (kb). The second ring indicates the positions of mutated genes in the genome (gray). The next five circles illustrate the five genomes used in this study (Newman D2C HOM [CP160002.1], Newman D2C [CP023391.1], Newman [AP009351.1], Newman HOM [CP160003.1], Newman UoM [NZ_LT598688.1]). The colored tiles inside each circle represent the positions of mutations within each respective genome.

Major bacterial laboratory strains are commonly shared between research laboratories and may differ substantially in their genome composition and phenotypic behavior from laboratory to laboratory, especially after years of storage and potential rounds of cultivation. The *S. aureus* Newman derivative used here (Newman HOM) originated from the Department of Microbiology at Trinity College Dublin, Ireland, and was transferred via the Universities of Geneva and Zurich, Switzerland, to the Institute for Medical Microbiology and Hygiene (IMMH) at Saarland University, Homburg, Germany. The *S. aureus* Newman D2C derivative (Newman D2C HOM) was obtained from the Institute for Medical Microbiology at Münster University, Germany, and transferred to IMMH in 2011. To test whether and how Newman HOM and Newman D2C HOM differ in their genetic repertoires from published Newman and Newman D2C genome sequences (i.e., GenBank accession numbers AP009351.1 [Newman], CP023390.1 [Newman NYU], NZ_LT598688.1 [Newman UoM], and CP023391.1 [Newman D2C]), we first determined the genome sequences of our Newman and Newman D2C derivatives by whole genome sequencing and compared these sequences with the Newman and Newman D2C genomes sequences published by Baba et al.^51^, Sause et al.^44^, and Monk et al.^52^. In line with our hypothesis that our Newman and Newman D2C derivatives probably differ in their genome composition from those used by Baba and colleagues^51^, Sause et al.^44^ and Monk et al.^52^, respectively, we observed a couple of single nucleotide polymorphisms (SNPs) and gene alterations in the genomes of Newman HOM and Newman D2C HOM that were not seen in the genome sequences reported for Newman, Newman NYU, Newman UoM, and Newman D2C, respectively (Fig. 1 and Supplementary Table S1). Notably, when directly compared to the above-mentioned reference sequences, both strain Newman HOM and Newman D2C HOM were most similar to strain Newman UoM at the genome level (Fig. 1). When checking for the mutations in the response regulator genes *agrA* and *saeR* identified in the Newman D2C reference genome sequence (GenBank accession no. CP023391.1), we also observed the *saeR*^C595T^ mutation in our Newman D2C derivative that was reported to render the *sae* system nonfunctional^44^.

**Fig. 2:**
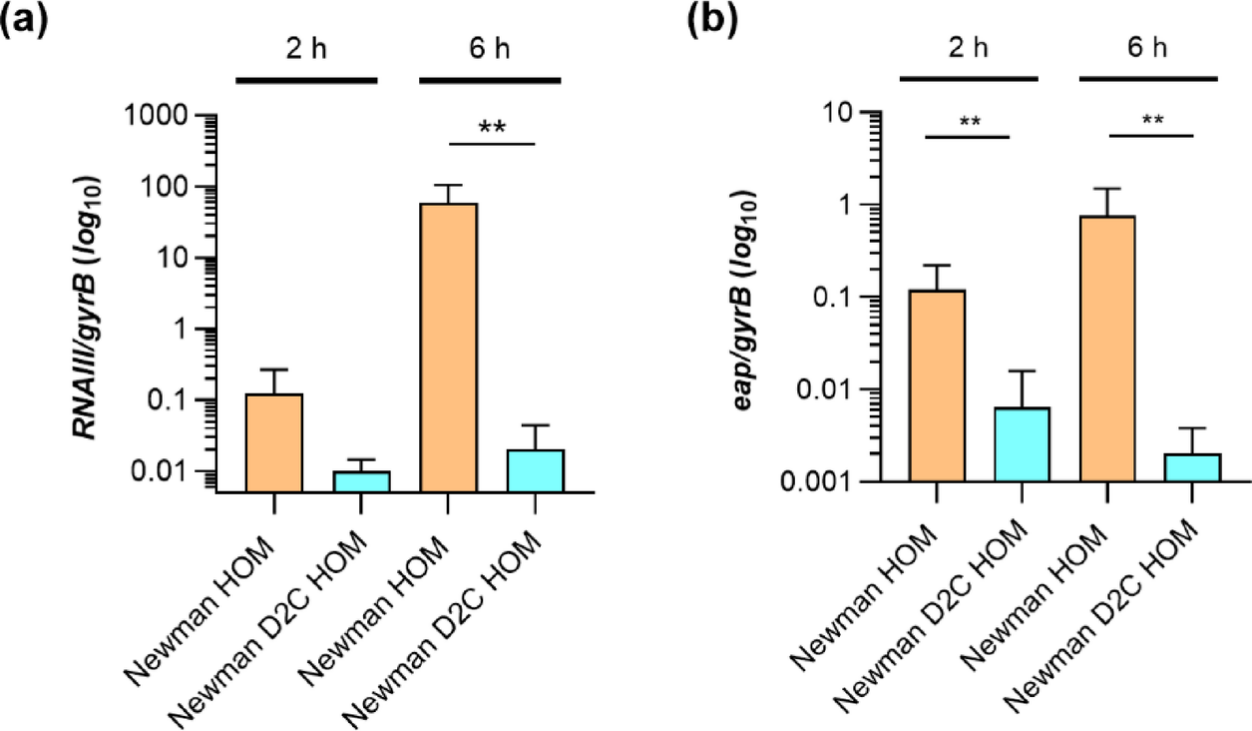
Quantitative transcript analyses of *RNAIII* and *eap* by qRT-PCR in Newman HOM (orange bars) and Newman D2C HOM (turquoise bars) cells grown in TSB at 37 °C and 225 rpm. (**a**,**b**) Transcript rates of *RNAIII* (**a**) and *eap* (**b**) at the time points indicated. Transcript rates were quantified in reference to the transcription of gyrase B (in copies per copy of *gyrB*). Data are presented as mean + SD of six biological replicates. **p < 0.01 (Mann–Whitney *U* test between Newman HOM and Newman D2C HOM at a given time point).

However, instead of the *agrA*^C168A^ SNP reported by Sause et al.^44^ for Newman D2C leading to a stop mutation at AgrA^[Y56^, we found a 1510 nucleotides-spanning, slightly truncated IS*1181* insertion sequence^53^ flanked at its ends by the 8-bp direct repeat AAATTATA in *agrA* at position A70. The reason why this IS element is stably integrated into the *agr* locus of Newman D2C HOM might be due to the fact that the transposase gene encoded by this IS element harbors a frameshift mutation at nucleotide A92 leading to a truncated open reading frame (ORF) encoding for 38 amino acids instead of the 439 amino acids reported for the transposase ORF of IS*1181*^53^. To confirm that the insertion/mutation in *agrA* and *saeR* found in Newman D2C HOM affect the activities of both two-component system response regulators, we tested for the transcription of *RNAIII*, the effector molecule of the *agr* locus that is transcriptionally controlled primarily by AgrA^54^, and for *eap*, encoding a secreted adhesin with immunomodulating functions that is under transcriptional control of SaeR^55^ (Fig. 2).

Upon culture in tryptic soy broth (TSB) under aerobic conditions (225 rpm and a culture to flask volume of 1:10), substantial transcript rates of *RNAIII* and *eap* were observed in strain Newman HOM that were particularly evident after 6 h of growth. In strain Newman D2C HOM, on the other hand, transcript rates for both genes were very low at both time points tested (Fig. 2), confirming that AgrA and SaeR activities are affected by the mutations found in the corresponding genes in Newman D2C HOM. Given the importance of *agr* and *sae* for virulence factor synthesis in *S. aureus*, one can also safely conclude that the Newman D2C HOM derivative is restricted in its capacity to express a large number of secreted virulence factors needed for full infectivity of *S. aureus*, which is in line with the observations made by Sause et al.^44^ for their Newman D2C derivative.

### Growth behavior of strains Newman HOM and Newman D2C HOM in batch culture

**Fig. 3:**
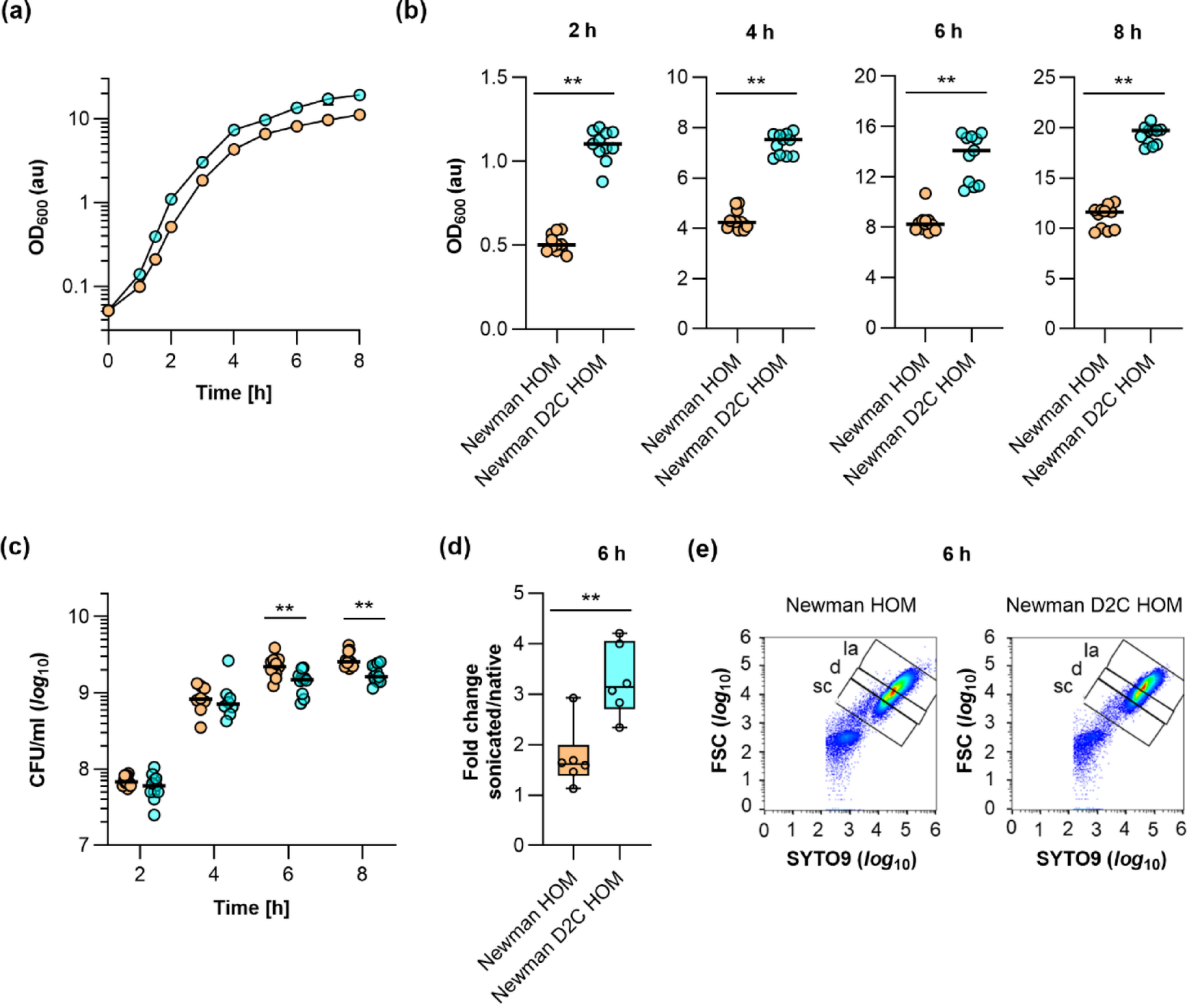
Growth kinetics of strains Newman HOM and Newman D2C HOM under aerobic conditions in tryptic soy broth (TSB). Bacteria were inoculated to an optical density at 600 nm (OD_600_) of 0.05 in TSB and cultured aerobically at 37 °C and 225 rpm in a culture-to-flask volume of 1:10. OD_600_ measurements of the cultures were determined hourly. Samples were diluted in TSB when an OD_600_ value of 0.8 was reached. The dilution factor was used to multiply with the measured result for the OD_600_ values displayed in the graphs. Calculated values are given as arbitrary units (au). (**a**) Growth kinetics of Newman HOM (orange symbols) and Newman D2C HOM (turquoise symbols) cell suspensions. The results are the mean ± SD of nine biological replicates. (**b**,**c**) OD_600_ values (**b**) and colony forming units (CFU) rates per ml (**c**) of the cell cultures at 2, 4, 6, and 8 h of growth, respectively. The data represent the values of every individual OD_600_ reading/CFU count (symbols) and the median (horizontal line). (**d**) Fold changes in the CFU rates per ml of Newman HOM and Newman D2C HOM 6 h cultures upon sonication. Data are presented as box and whisker plots (min-to-max). Symbols indicate the mean values per experiment (*n* = 6). (**e**) Forward scatter (FSC) versus green fluorescence cytograms of SYTO9-stained Newman HOM and Newman D2C HOM 6 h cultures (diluted 1:100 in PBS). Data shown represent one of the assays carried out in three biological replicates. *sc* single cells, *d* doublets, *la* larger aggregates. **p < 0.01 [Mann–Whitney *U* test (**b**,**d**) and Kolmogorov–Smirnov test with two-stage linear step-up procedure of Benjamini, Krieger, and Yekutieli (**c**)].

When Newman HOM and Newman D2C HOM overnight (o/n) cultures were prepared in TSB, much higher optical densities at 600 nm (OD_600_) values for the Newman D2C HOM cultures were repeatedly observed at the next morning. This suggests that strain Newman D2C HOM can produce more biomass under these growth conditions than strain Newman HOM. To better display this growth difference between strains Newman HOM and Newman D2C HOM, we monitored the growth behavior of both strains in TSB batch cultures under aerobic growth conditions for up to eight hours (Fig. 3).

In line with the overnight culture findings, we observed significantly higher OD_600_ readings for strain Newman D2C HOM cultures that were present already after 2 h of growth (Fig. 3b), suggesting that Newman D2C HOM cells can multiply faster under these conditions. To substantiate this hypothesis, we determined the generation times of both strains during the exponential growth phase (i.e., 1–3 h) and transition phase (i.e., 4–6 h, Table 1).

**Table 1:** Generation times of exponential growth phase cells and transition phase cells of *S. aureus* strains Newman HOM and Newman D2C HOM cultivated in TSB under aerobic conditions.

Strain	Generation time (min)*	*p* value^#^
Exponential growth phase (1–3 h)		
Newman HOM	28.54 ± 0.38	
Newman D2C HOM	26.81 ± 0.75	< 0.0001
Transition phase (4–6 h)		
Newman HOM	126.1 ± 10.08	
Newman D2C HOM	145.9 ± 26.12	0.2973

*Data are presented as mean ± SD (*n* = 9).

^#^*p*-values were determined by Mann–Whitney *U* test.

This analysis revealed that Newman D2C HOM cells displayed a significantly shorter generation time than Newman HOM cells during exponential growth, while there was no clear difference in the generation times between both strains in the transition phase. However, when colony forming units (CFU) rates/ml of the TSB cultures were determined (Fig. 3c), rather comparable CFU/ml rates were observed for both strains at 2 and 4 h, respectively. Notably, at 6 h and 8 h, CFU/ml rates were even smaller for the Newman D2C HOM cell cultures, which was in striking contrast to the OD_600_ readings that suggested higher bacterial cell densities in the Newman D2C HOM cell cultures also at the later growth stages. One possible explanation for this discrepancy in OD_600_ readings and CFU rates seen with the Newman D2C HOM cell cultures might be that Newman D2C HOM cells formed more or larger micro-aggregates under these growth conditions than Newman HOM, which would still yield in an increase in OD_600_ readings but result in fewer CFU rates per ml. To test this hypothesis, we compared the CFU rates of 6 h cultures of strains Newman HOM and Newman D2C HOM that were determined before and after sonication (Fig. 3d). While sonication of the Newman HOM 6 h cultures only moderately increased the CFU numbers per ml (< 2-fold), a significantly higher increase in CFU rates (> 3-fold) was observed when the Newman D2C HOM 6 h cultures were sonicated. To further substantiate our hypothesis, 6 h TSB cultures of Newman HOM and Newman D2C HOM were supplemented with the fluorescent dyes SYTO9 (which stains DNA of viable and dead cells) and propidium iodide (PI, which only stains DNA of cells with disrupted cytoplasmic membranes) and subjected to fluorescence activated cell sorting (FACS). In line with our hypothesis, we observed a shift to the upper right area for the SYTO9-stained Newman D2C HOM cell population in the SYTO9 intensity fluorescence flow cytometry density plots (Fig. 3e and Supplementary Fig. S1), confirming a higher proportion of larger micro-aggregates in the 6 h TSB cell cultures of strain Newman D2C HOM (52.8 ± 2.4%), when compared to Newman HOM (37.2 ± 3.9%). Notably, micro-aggregates formed by Newman D2C HOM after 6 h of growth in TSB also tended to dissolve less efficiently upon sonication than those produced by Newman HOM (see Supplementary Fig. S1d,f). A probable effect of differences in cell viability between the Newman HOM and Newman D2C HOM 6 h cultures could be largely excluded, as both cultures contained comparable proportions of PI-stainable cells, which were for the bacterial cell cultures of both strains at this growth stage below 2% (Supplementary Fig. S2). Both findings indicate that the CFU rates obtained with Newman D2C HOM cultures at the later growth stages are probably undervalued due to a larger degree of micro-aggregate formation by this strain.

### *S. aureus* strains Newman HOM and Newman D2C HOM differ in their capacities to adhere to abiotic surfaces

**Fig. 4:**
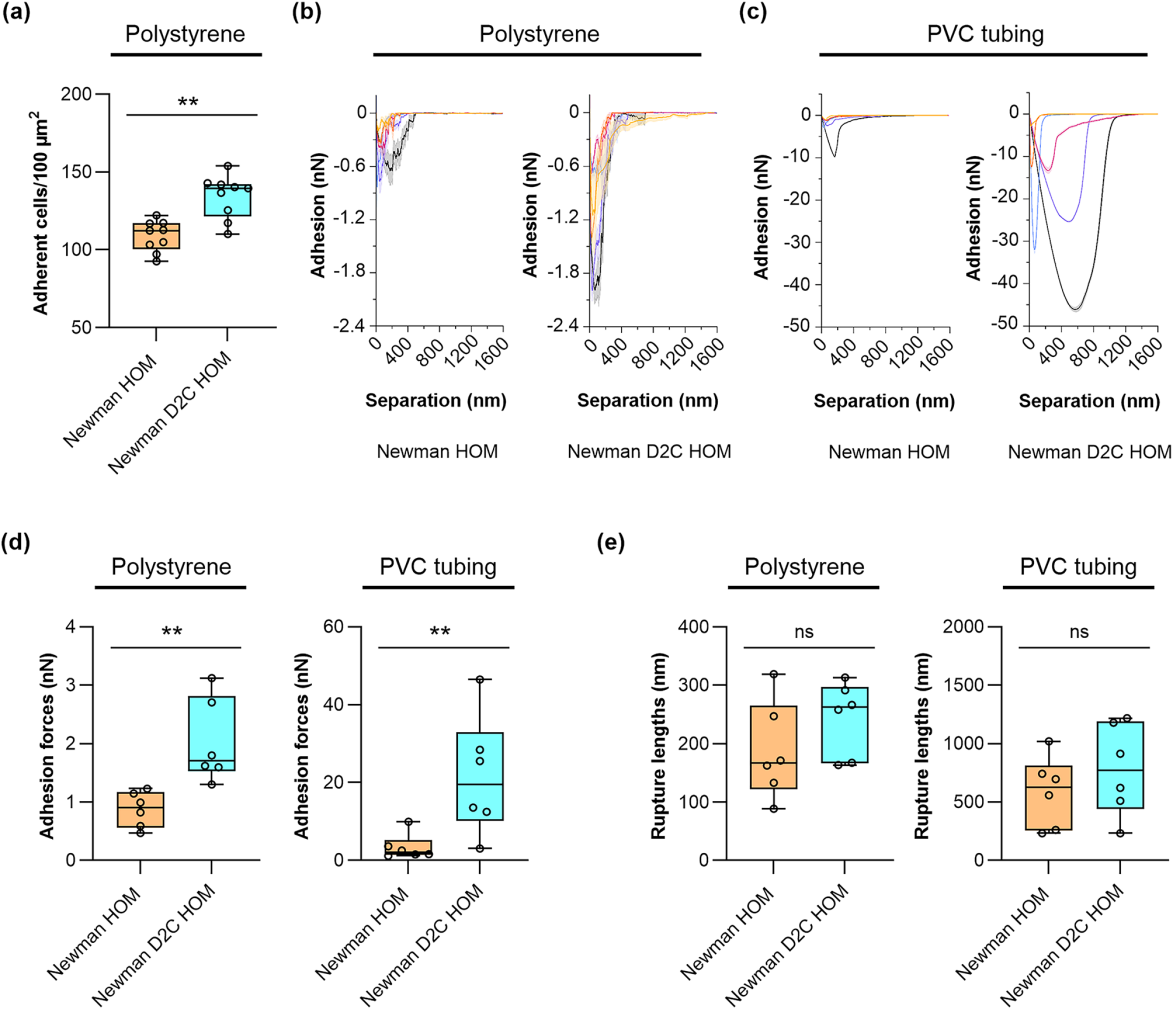
Quantitative and qualitative adhesion capacities of strains Newman HOM and Newman D2C HOM on artificial surfaces. (**a**) Quantitative adhesion of cells of strains Newman HOM (orange filled box and symbols) and Newman D2C HOM (turquoise filled box and symbols) on polystyrene-based 96-well microplates. Exponential growth phase cells were fluorescence-labeled with Hoechst 33342 and placed into the wells of the Nunclon Delta-treated, flat-bottom 96-well microplate. Unbound and loosely bound bacterial cells were removed by washing, and adherent cells were counted by fluorescence microscopy. Box and whisker plots (min-to-max) of the mean values of adherent bacteria found in a 100 µm^2^ area (*n* = 9 images/strain obtained by three biological experiments). (**b–e**) Adhesive strengths of cells of strains Newman HOM and Newman D2C HOM on polystyrene and PVC tubing. (**b**,**c**) Mean SCFS retraction curves of six individual bacteria (indicated by different colors) per strain on polystyrene (**b**) and PVC tubing (**c**), respectively. Bacterial cells were probed with the substratum with a 5 s surface delay time. The means were calculated using 30 force-distance curves per cell and surface type (shaded areas represent the standard deviations per bacterial cell). (**d**,**e**) Box and whisker plots (min-to-max) of the mean adhesion forces (**d**) and mean rupture lengths (**e**) of cells of strains Newman HOM and Newman D2C HOM probed on polystyrene and PVC tubing, respectively. Mean values of the individual cells are indicated as round symbols. *ns* not significant; **p < 0.01 (Mann–Whitney *U* test).

Our efforts to compare the in vitro biofilm formation capacities of strains Newman HOM and Newman D2C HOM started with the determination of the initial adhesion characteristics of both strains towards polystyrene (PS) and polyurethane (PU). The former synthetic polymer served as surrogate for our static and dynamic 96-well microplate assays. PU was chosen, as PU-based PVC tubing served as seeding ground for the bacteria in our flow system and the foreign body-related in vivo model. Placing fluorescence-stained exponential growth phase cells of strains Newman HOM and Newman D2C HOM into the wells of Nunclon Delta-treated, flat-bottom 96-well microplates revealed that Newman D2C HOM cells adhered in significantly larger quantities to this kind of plastic surface than cells of strain Newman HOM (Fig. 4a), suggesting that Newman D2C HOM cells display a higher affinity towards this kind of substratum.

Atomic force microscopy-based single-cell force spectroscopy (SCFS) was utilized next to determine the adhesion forces between the bacterial cells on polystyrene and PU-based PVC tubing, respectively (Figs. 4b–e). These studies identified significantly higher adhesion forces for cells of strain Newman D2C HOM on both types of surfaces, the hydrophilic polystyrene (advancing water contact angle of 74.30 ± 1.73°) and the hydrophobic PU-based PVC tubing (advancing water contact angle of 105.73 ± 2.59°) when compared to the cells of strain Newman HOM (Fig. 4b,c). The superior adhesion capacities of cells of strain Newman D2C HOM were particularly evident on the PVC tubing and about one order of magnitude higher than those recorded with Newman HOM cells on this kind of surface (Fig. 4d). However, with respect to the retraction curve wave forms (Fig. 4b,c), both strains displayed rather comparable retraction curve characteristics that are commonly found for *S. aureus* on hydrophobic and hydrophilic surfaces, respectively^7,16^. Similarly, cells of both strains did not differ markedly in their rupture lengths (i.e., the maximum distance needed to detach the bacterial cell from its substratum completely) on both types of surfaces (Fig. 4e). These findings suggest that both strains probably express a rather comparable repertoire of adhesion molecules but likely differ in the amounts of adhesion factors presented on the bacterial cell surface.

### *S. aureus* strains Newman HOM and Newman D2C HOM differ significantly in their capacities to form biofilms under static and dynamic conditions in 96-well microplates

**Fig. 5:**
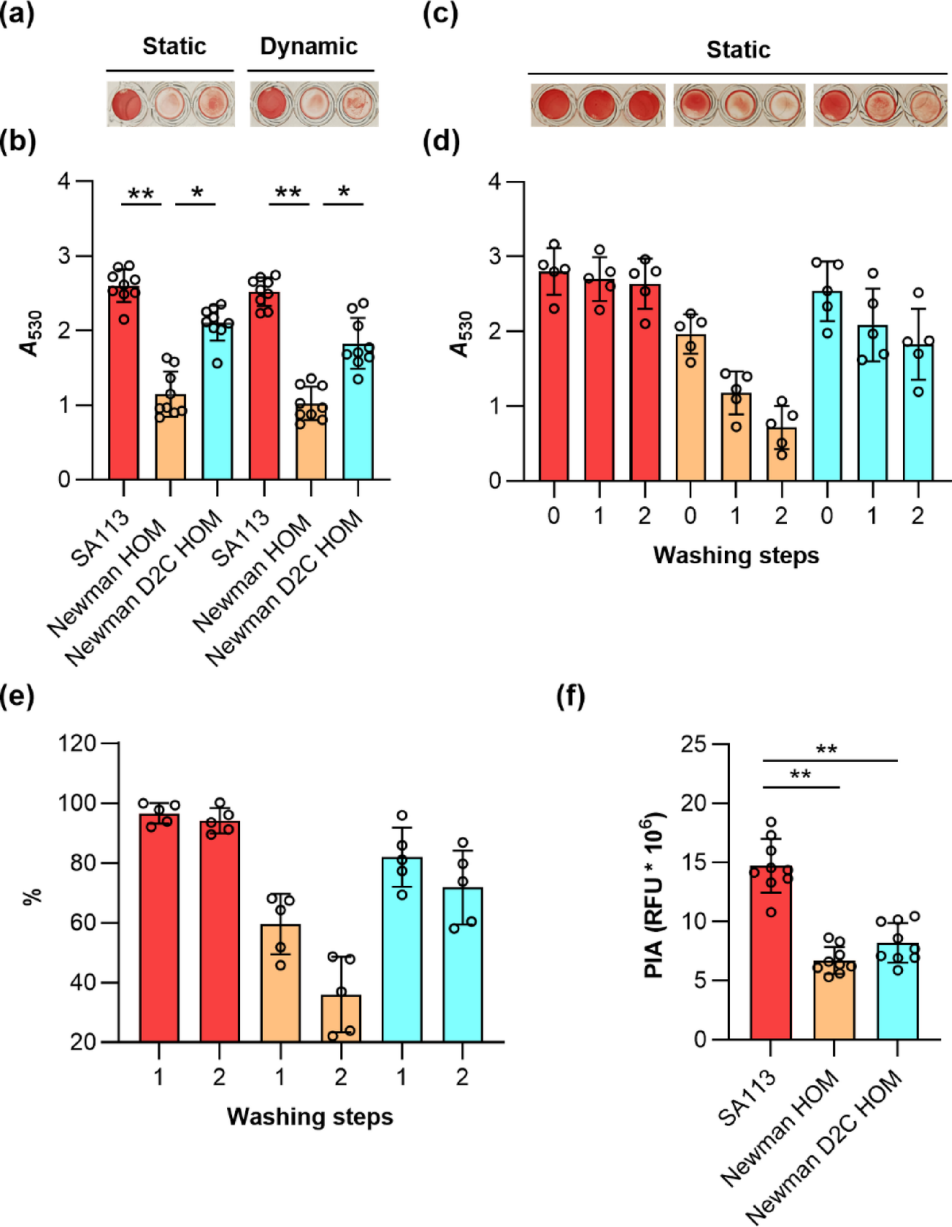
Biofilm formation of *S. aureus* strains SA113, Newman HOM, and Newman D2C HOM in TSB supplemented with 0.5% glucose. Exponential growth phase cells of strains SA113 (red bars), Newman HOM (orange bars), and Newman D2C HOM (turquoise bars) were inoculated into TSB supplemented with 0.5% glucose (TSB-G) and cultured in Nunclon Delta-treated 96-well microplates for 18 h at 37 °C under static or dynamic (120 rpm) conditions as indicated. (**a–d**) Vegetation were washed twice prior to the safranin staining (**a**,**b**) or washed as indicated (**c**,**d**). (**a**,**c**) Representative images of safranin-stained vegetation. (**b**,**d**) *A*_530_ readings of safranin contents in the wells after solubilization with 30% acetic acid. (**e**) Impact of the washing steps on the *A*_530_ readings of safranin contents in the wells. Values are given in relation to the safranin signals seen in wells that were not washed, which were set to 100%. (**f**) PIA contents of the vegetation formed by strains SA113, Newman HOM, and Newman D2C HOM in TSB-G in 96-well microplates cultured for 18 h at 37 °C under static conditions. PIA contents of the biofilms were determined by staining the vegetation with XFD488-labeled WGA (2.5 µg/ml) and determining the fluorescence signals of the incorporated dye at 480/530 nm (Excitation/Emission). Differences in PIA contents are given as relative light units (RFUs). Results represent the averages of five to nine independent experiments done in duplicate. Error bars indicate the standard deviation of the mean. Round symbols indicate the mean *A*_530_/%/RFU values of individual experiments. *p < 0.05; **p < 0.01 (Kruskal–Wallis test and Dunn’s multiple comparison test).

Given the increased adhesion capacities noticed for strain Newman D2C HOM in our SCFS studies, we wondered whether this adhesion phenotype also translates into a stronger biofilm formation capacity of this strain. Thus, we first tested the capacities of strains Newman HOM and Newman D2C HOM to form biofilms in 96-well PS-based microplates in glucose-supplemented TSB (TSB-G), a very frequently used growth condition in this kind of assay. In this series of experiments, we also included strain SA113 for comparison, which is a strong PIA-dependent biofilm-forming *S. aureus* strain under these growth conditions^21^. When exponential growth phase cells of all three strains were seeded into flat-bottom wells of Nunclon Delta-treated 96-well microplates and cultured in TSB-G for 18 h at 37 °C under static or dynamic (120 rpm) conditions, followed by washing and safranin staining steps, strain SA113 displayed the highest absorption rates at 530 nm (*A*_530_), suggesting that this strain formed a strong biofilm under these growth conditions that was not detached by washing steps (Fig. 5a,b).

Substantial *A*_530_ readings were also obtained for strain Newman D2C HOM under these growth conditions, also they did not approach the levels yielded by strain SA113, which is in line with earlier findings^21^. In contrast, safranin-stained biofilms formed by strain Newman HOM under these test conditions produced much lower *A*_530_ values, which were significantly smaller than those seen with strains SA113 and Newman D2C HOM, respectively. However, when washing the biofilms formed after 18 h of growth on the bottom of the wells, we repeatedly noticed that most of the biomass formed by strain Newman HOM on the well bottom was detached from the surface and removed with the washing. The latter observation suggests that strain Newman HOM produces a biofilm under these growth conditions that is more loosely attached to the surface or that most of the biomass seen on the bottom of the well after removal of the growth medium originated from cells that sedimented over time and did not form an extracellular matrix.

To test the capacities of the three strains to retain their biomasses on the surface of the well bottoms upon washing, we either air-dried and safranin-stained the biomasses formed on the bottom of the well after removal of the growth medium (0 washing steps) or washed the wells once and twice, respectively, before the remaining biomasses were air-dried and safranin-stained (Fig. 5c–e). This test procedure revealed that the biomasses formed by SA113 on the bottoms of the wells were almost completely retained even after two washes (94.1 ± 4.2% of the safranin signals seen in wells that were not washed). Safranin-stained Newman D2C HOM biofilms lost under these test conditions about 1/4 to 1/3 of the staining signal (71.8 ± 12.3% of the safranin signal seen in wells that were not washed; Fig. 5e). Safranin-stained Newman HOM biofilms, however, displayed after two washes *A*_530_ readings that were 35.9 ± 12.7% of the safranin signals seen in wells that were not washed. Thus, the washing steps included in this assay removed roughly 2/3 of the biomass of strain Newman HOM formed on the well bottom after 18 h of growth in TSB-G under static conditions. These findings suggest to us that most of the biomass produced by strain Newman HOM under these growth conditions was derived from planktonic cells that sedimented to the bottom of the well over time, which were not embedded into an ECM and were therefore more easily removed by the washing steps. Alternatively, strain Newman HOM might form a biofilm in TSB-G that is more loosely attached to the PS-based surface than those formed by strains SA113 and Newman D2C HOM, respectively. The high *A*_530_ readings seen with strain SA113 in this assay after two washes indicate, on the other hand, that the biomass formed by this strain after 18 h of growth in TSB-G consists mostly of cells embedded in an ECM and, if at all, only of a small proportion of sedimented planktonic cells (< 10%) being accessible to removal by the washing procedure.

To get an idea about the ECMs produced by strains Newman HOM and Newman D2C HOM under these growth conditions, the 18 h old TSB-G grown vegetation formed on the well bottoms were stained with a fluorophore-labeled wheat germ agglutinin (WGA). This carbohydrate-binding lectin has a high affinity for *N*-acetylglucosamine moieties and can be used to stain the PIA content in the ECM of staphylococcal biofilms^56^. We first made use of XFD488-labeled WGA to quantify the PIA contents in the biomasses produced by strains Newman HOM, Newman D2C HOM, and SA113 on the bottoms of the wells (Fig. 5f). In line with earlier reports, we observed strong fluorescence signals in wells inoculated with SA113, while wells inoculated with Newman HOM and Newman D2C HOM produced significantly smaller fluorescence signals under these test conditions, respectively. These findings support the idea that SA113 produces strong PIA-dependent biofilms in this type of assay, while biofilm formation of strain Newman D2C is less dependent on PIA^21,47^ The latter statement is further substantiated when taking into account, that Newman HOM vegetation formed on the bottoms of the wells produced comparable fluorescence signals as Newman D2C HOM (6.7 × 10^6^ ± 1.1 × 10^6^ vs. 8.2 × 10^6^ ± 1.7 × 10^6^ RLUs per well; *p* = 0.6049 [Kruskal–Wallis test and Dunn’s multiple comparison test]), albeit of losing more biomass than the latter strain during the washing steps included in this assay.

### *S. aureus* strains Newman HOM and Newman D2C HOM also differ significantly in their capacities to form biofilms in TSB-G under static conditions in 6-well microplates

**Fig. 6:**
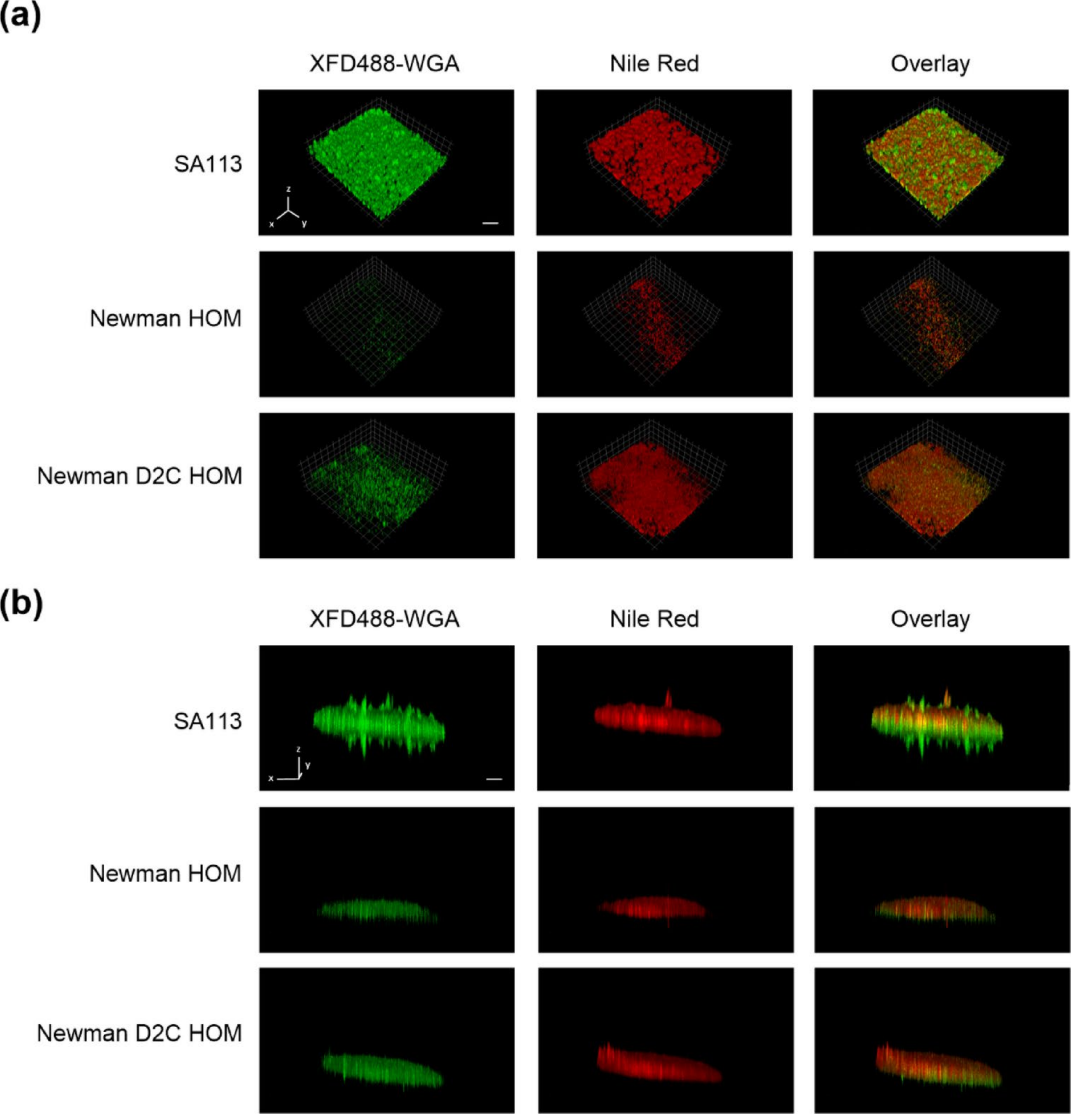
Three-dimensional organization and PIA distribution of the vegetation formed by strains SA113, Newman HOM, and Newman D2C HOM in PS-based microplates. Exponential growth phase cells of *S. aureus* strains were inoculated into TSB supplemented with 0.5% glucose and cultured in tissue culture-treated, PS-based 6-well microplates for 18 h at 37 °C under static conditions. The vegetation formed were stained with XFD488-WGA and Nile Red (NR), and fluorescence was monitored with CLSM. (**a**,**b**) Three-dimensional image reconstructions of *z* series were recorded at 525 nm (XFD488) and 595 nm (NR), respectively. Reconstructed top view (**a**) and side view (**b**) images of the vegetation formed by the test strains. CLSM reconstructions are representative of three separate experiments. Each side of a grid square in the image reconstructions represents 100 μm. Scale bar, 200 μm (scale bar applies to all images in the respective panel).

To learn more about the three-dimensional (3D) organization of the vegetation formed on the well bottoms and the spatial distribution of PIA, we grew biofilms of strains Newman HOM, Newman D2C HOM, and SA113 in tissue culture-treated, PS-based 6-well microplates for 18 h. Following this, the vegetation were washed once with PBS and the remaining biomasses stained with XFD488-labeled WGA and the fluorescent dye Nile Red (NR), a lipophilic stain that can be used to stain *S. aureus* membranes^57^. When fluorophore-labeled biofilms were monitored by confocal laser scanning microscopy (CLSM), we observed a rather characteristic 3D structure for strain SA113 (Fig. 6).

Strain SA113 formed multiple towers in between a dense layer of bacterial cells that was both positive for XFD488-labeled WGA demonstrating PIA localization, and Nile Red (NR) demonstrating localization of bacterial cells (Figs. 6a,b). This steric order of cells and PIA was seen neither with strain Newman D2C HOM nor with strain Newman HOM. The former strain produced, under these conditions, dense vegetation on the bottom of the well that were mostly positive for NR. Stained vegetation of strain Newman HOM, on the other hand, appeared as patchy structures with only little biomass remaining on the surface of the well bottom after the washing and staining steps included in this assay format. Reconstructed side view images of the vegetation formed by the test strains revealed the thickest stained areas for strain SA113, followed by strain Newman D2C HOM and Newman HOM, respectively (Fig. 6b), a finding that is in line with our observations made with these strains in our 96-well microplate biofilm assays described above (Fig. 5). Taken together these observations indicate strain SA113 to form a thick and structured biofilm in TSB-G under static conditions. Strain Newman D2C HOM forms a slightly thinner and evenly distributed biofilm, while strain Newman HOM produces a thin and fragmentary vegetation with most of the biomass formed being removed by the washing and staining steps (Fig. 6a).

### *S. aureus* strains Newman HOM and Newman D2C HOM differ significantly in their capacities to form biofilms in presence of human blood serum

**Fig. 7:**
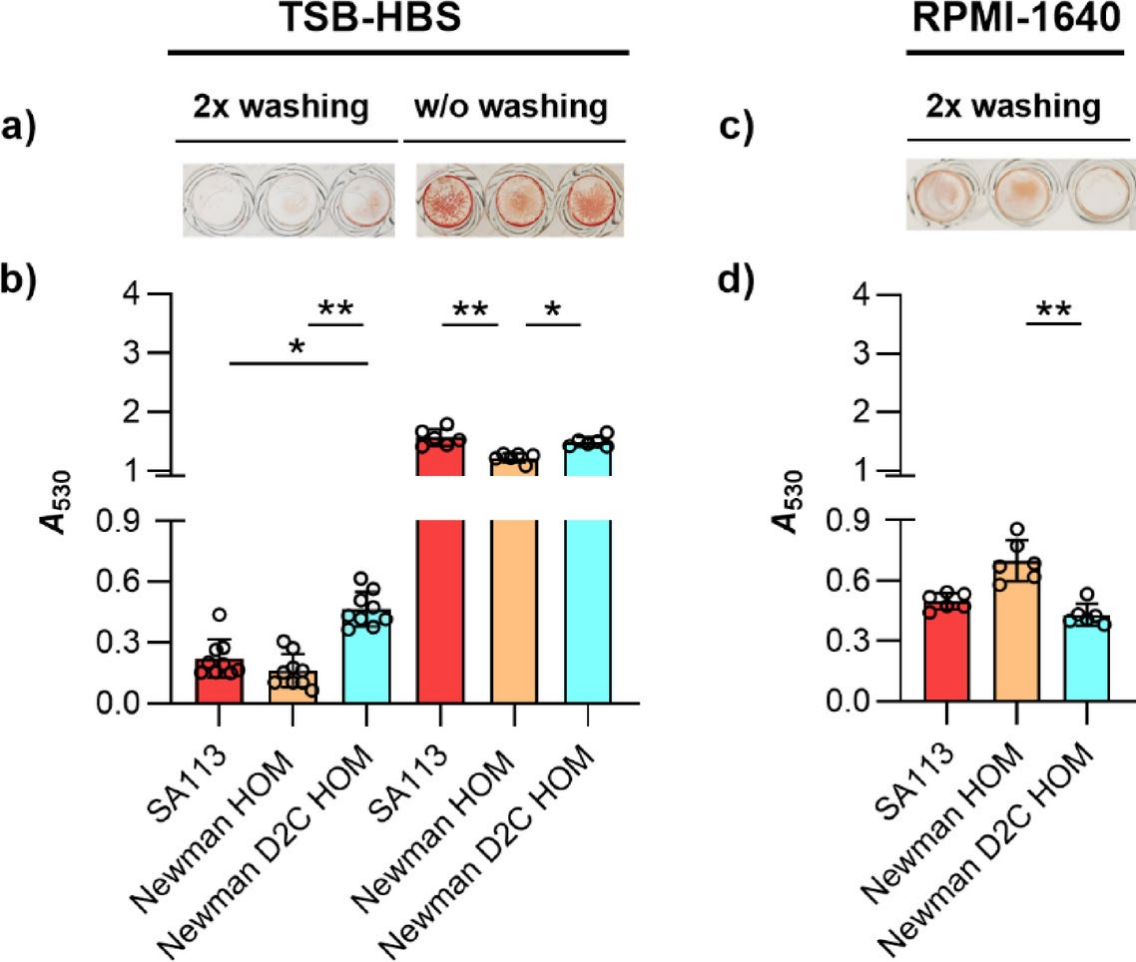
Biofilm formation of *S. aureus* strains SA113, Newman HOM, and Newman D2C HOM in TSB-HBS or RPMI-1640 under static conditions. (**a**,**b**) Exponential growth phase cells of strains SA113 (red bars), Newman HOM (orange bars), and Newman D2C HOM (turquoise bars) were inoculated into TSB supplemented with 5% human blood serum (TSB-HBS; **a**) or Roswell Park Memorial Institute 1640 medium (RPMI-1640; **b**) and cultured in Nunclon Delta-treated 96-well microplates for 18 h at 37 °C under static conditions. Washing steps prior to the safranin staining were performed as indicated. (**a**,**c**) Representative images of safranin-stained vegetation. (**b**,**d**) *A*_530_ readings of safranin contents in the wells after solubilization with 30% acetic acid. Results represent the averages of six to nine independent experiments done in duplicate. Error bars indicate the standard deviation of the mean. Round symbols indicate the mean *A*_530_ values of individual experiments. *p < 0.05; ** *p* < 0.01 (Kruskal–Wallis test and Dunn’s multiple comparison test).

To test whether larger differences in biofilm formation between strains Newman HOM and Newman D2C HOM might also be visible in other growth settings, the capacities of both strains to form vegetation on the bottoms of 96-well micro-plates in TSB containing 5% human blood serum (TSB-HBS; Fig. 7a,b) was determined. This growth condition was of particular interest for us since it closer approaches the clinical situation, and since biofilm formation of strains Newman D2C and SA113 were reported to rely largely on Eap in this setting^47^. Given that Newman D2C is a *saeR* mutant and that *eap* transcription is strongly affected by the *sae* system in *S. aureus*^55^, this strain produces only very little Eap, which is also confirmed here on the transcriptional level (see Fig. 2b). Strain Newman, on the other hand, is a strong Eap producer due to its *saeS*^*P*^ allele^58,59^, a phenotype that was also reproduced in our transcriptional studies (Fig. 2b). Thus, we particularly wondered how these differences in *eap* expression observed between strains Newman HOM and Newman D2C HOM translate into biomass production when the test strains were cultured in the presence of 5% HBS.

When compared to the biofilms formed in TSB-G under static conditions (Fig. 5a,b), all three strains produced clearly lower vegetation in TSB-HBS that remained on the well bottoms after washing and staining (Fig. 7a). This phenomenon was particularly evident for the biofilms formed by strain SA113, which displayed *A*_530_ readings that were in the range of those observed with strain Newman HOM, and significantly lower as those determined for strain Newman D2C HOM (Fig. 7b). These observations are surprising in several ways. Thompson and colleagues^47^ reported for comparable assay conditions an increase in biofilm formation of strain SA113 in presence of serum, and that strain SA113 forms larger vegetation than strain Newman D2C under these conditions. A positive effect of liquid blood components on *S. aureus* biofilm formation was also suggested by Cardile et al.^60^, who reported an increased biofilm formation of different *S. aureus* strains in the presence of human plasma. Notably, a supporting effect of plasma on biofilm formation was also reported for *Staphylococcus epidermidis* by Skovdal et al.^61^, who also noted, however, that this effect was not visible in the standard 96-well microplate assay due to poor binding of the bacterial aggregates to the bottoms of the wells that were formed by *S. epidermidis* in presence of plasma. Assuming that this may also hold truth for *S. aureus*, we next determined the *A*_530_ values of biofilms formed by strains SA113, Newman HOM, and Newman D2C HOM on the bottoms of the microplate wells after 18 h of growth in TSB-HBS, however, this time without washing the biofilms before the staining procedure (Fig. 7). Similar to our observations made with our strain set in TSB-G (Fig. 5c,d), we observed again clearly higher *A*_530_ values in the wells inoculated with strains Newman HOM and Newman D2C HOM, respectively, when the vegetation formed were directly air-dried and safranin-stained after the removal of the growth medium (w/o washing). Notably, this phenotype was also observed for the biofilms formed by SA113 in TSB-HBS, while this was not the case for this strain in TSB-G, in which SA113 produced biofilms that were virtually not removed from the surface by the washing steps prior to staining (Fig. 5d). Particularly the findings made with strain SA113 in TSB-HBS support our assumption that *S. aureus* produces in presence of HBS vegetation that are less strongly bound to the surface than those formed in TSB-G.

When comparing the *A*_530_ values observed for all strains in TSB-HSB without washing to the *A*_530_ readings observed in TSB-G after this procedure, about 2- to 3-fold lower *A*_530_ readings were determined in TSB-HBS than in TSB-G. The latter phenotype is probably due to the fact that the biofilm formation of *S. aureus* depends in this assay type largely on the amount of glucose in the growth medium^62^, although we cannot exclude the possibility that the biofilms formed by all three strains in TSB-HBS are so loosely attached to the surface that substantial parts of the biomasses formed were already removed along with the growth medium.

The direct comparison of the *A*_530_ readings of safranin-stained biofilms formed by Newman HOM and Newman D2C HOM in TSB-HBS revealed significantly higher values for the low-level Eap producer Newman D2C HOM (Fig. 7b), suggesting that the amount of Eap produced by *S. aureus* is probably not a major factor for the biofilm formation capacity of this species. The superior capacity of strain Newman D2C HOM to form biofilms in TSB-HBS that are resistant to the washing steps might be due to its capacity to adhere stronger to the polystyrene surface than strain Newman HOM, as demonstrated for individual cells in our SCFS studies (Fig. 4). A potential effect of the *agrA* mutation found in Newman D2C HOM on the biofilm formation phenotype in TSB-HBS can be largely excluded, as strain SA113 also harbors a mutation in the *agr* locus that renders this strain *agr* negative but does not show an enhanced biofilm formation capacity in TSB-HBS when compared to strain Newman HOM (Fig. 7b).

### *S. aureus* strains Newman HOM and Newman D2C HOM differ significantly in their capacities to form biofilms in RPMI-1640

To further approach a physiological growth setting, we tested the capacities of strains SA113, Newman HOM, and Newman D2C HOM to form vegetation on the bottoms of 96-well micro-plates in Roswell Park Memorial Institute 1640 medium (RPMI-1640). In this chemically defined mammalian cell culture medium *S. aureus* produces a global gene expression profile comparable to the one seen in human plasma^63^. Similar to our findings made with TSB-HBS, we observed again for all three strains lower vegetation in RPMI-1640 that remained on the well bottoms after washing and staining (Fig. 7c,d), when compared to the biofilms formed in TSB-G under static conditions (Figs. 5a,b). However, unlike seen with TSB-G and TSB-HBS (Figs. 5b and 7b), strain Newman HOM displayed the highest *A*_530_ readings in RPMI-1640 that were significantly higher than those determined for strain Newman D2C HOM (Fig. 7d). These findings indicate that the capacity of *S. aureus* to form a biofilm on the bottoms of 96-well microplates can vary greatly depending on the growth medium chosen, and that a high biofilm-forming capacity of a strain in medium A does not necessarily indicate a high biofilm-forming capacity of the same strain in medium B.

### The capacities of *S. aureus* strains Newman HOM and Newman D2C HOM to produce biofilms on PU-based catheter tubing differ depending on the culture medium

**Fig. 8:**
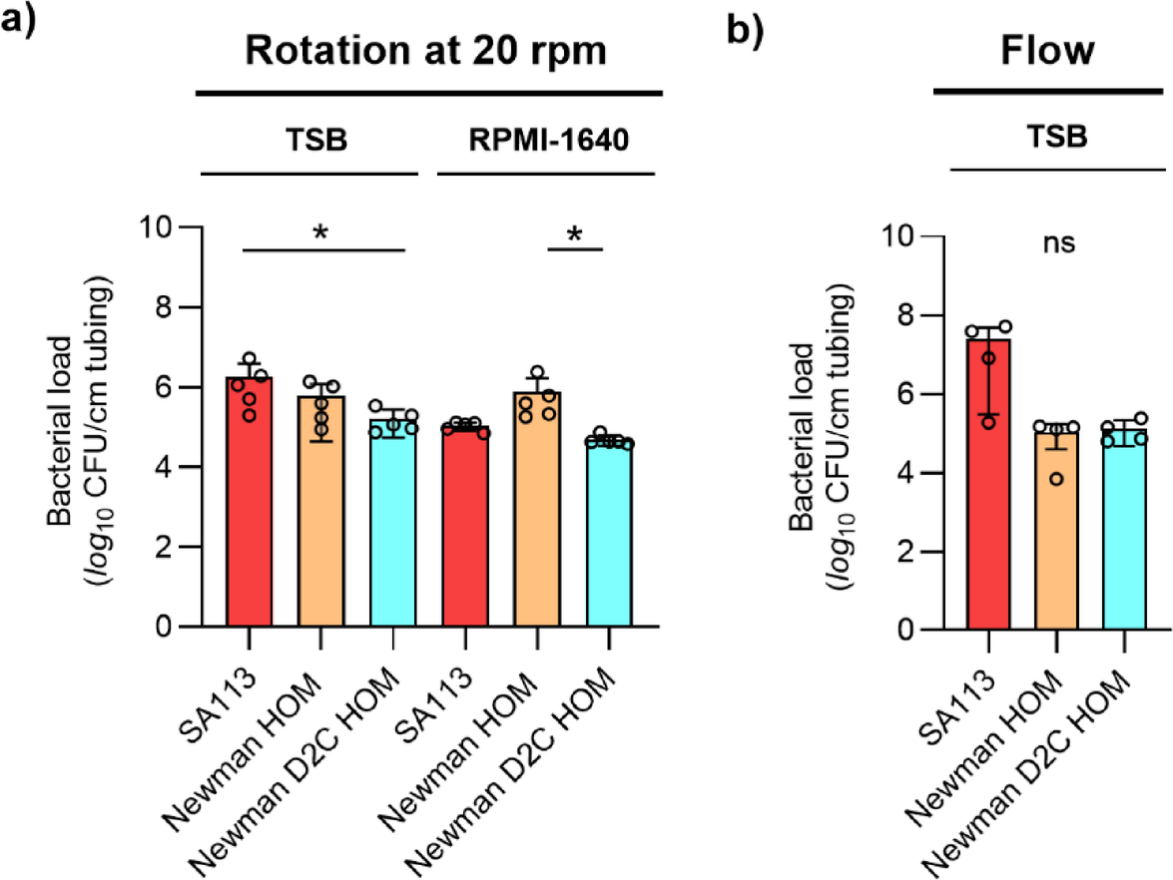
Biofilm formation of *S. aureus* strains SA113, Newman HOM, and Newman D2C HOM on PVC tubing under dynamic conditions. (**a**) Cell suspensions of strains SA113 (red bars), Newman HOM (orange bars), and Newman D2C HOM (turquoise bars) were used to inoculate the lumen of 1 cm long PU-based PVC tubing fragments, and the infected tubing fragments were cultured in TSB or RPMI-1640 for 5 days at 37 °C under non-nutrient limiting and dynamic conditions (rotation at 20 rpm). (**b**) Biofilm formation of *S. aureus* strains SA113, Newman HOM, and Newman D2C HOM on the lumen of PVC tubing in TSB under constant flow (1 ml/min). CFU rates of detached biofilms are shown. Error bars indicate the standard deviation of the mean. Round symbols indicate the CFU values of individual experiments. *ns* not significant; *p < 0.05 (Kruskal–Wallis test and Dunn’s multiple comparison test).

To test for the capacity of both strains to form biofilms on PU-based catheter tubing, two different assays were used: In the first assay, the lumen of PVC tubing fragments were infected and cultured under dynamic conditions (rotation at 20 rpm) in TSB and RPMI-1640, respectively. After five days of cultivation in TSB under non-limited nutrient conditions, strain SA113 produced the highest CFU numbers on the PVC tubing, which were in the mean 2.9 and 11-fold higher as those seen with Newman HOM and Newman D2C HOM, respectively (Fig. 8a). However, when cultured in RPMI-1640, Newman HOM produced the highest CFU numbers on the PVC tubing, which were significantly higher as those seen with Newman D2C HOM (Fig. 8a).

To test for the capacity of the strain set to form biofilms on PU-based catheter tubing under constant flow, half of the lumen of the PVC tubing was infected with the bacterial inoculum, and the PVC subsequently connected via tubing and a peristaltic pump to a medium reservoir. Fresh, pre-warmed TSB medium was channeled for 18 h through the PVC with a constant flow rate of 1 ml/min, thereby creating a low fluid shear stress of ~ 1 dyn/cm^2^. Under the latter conditions, strain SA113 produced a median vegetation of 2.35 × 10^7^ CFU per cm of tubing, while vegetation of strains Newman HOM and Newman D2C HOM were both 2-*log*_10_ lower and on a rather comparable level to each other (Fig. 8b). These findings illustrate once again that the biofilm formation capacity of individual *S. aureus* strains may vary substantially depending on the growth media, substrates and growth conditions chosen, and that the biofilm formation capacity of strain Newman HOM is superior over those of SA113 and Newman D2C HOM, when cultured in RPMI-1640.

### *S. aureus* strains Newman HOM and Newman D2C HOM produce comparable vegetation on catheter tubing in a murine foreign body-related infection model

**Fig. 9:**
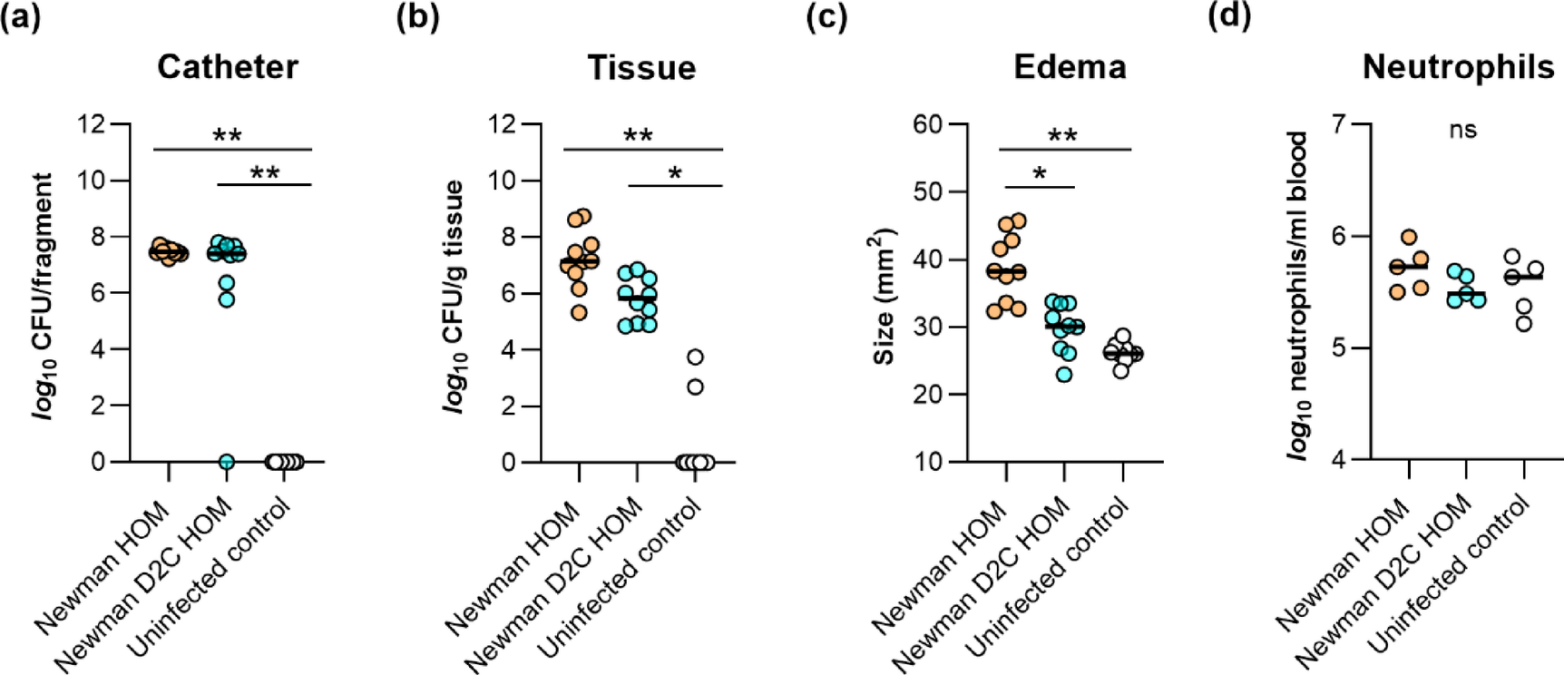
Infectivity of *S. aureus* strains Newman HOM and Newman D2C HOM in a murine foreign body-related infection model. Sterile polyurethane-based peripheral venous catheter tubing fragments were implanted subcutaneously into the left and right flanks of normoglycemic C57BL/6 mice (*n* = 5 per group) and inoculated with cells of *S. aureus* strain Newman HOM (orange symbols) and Newman D2C HOM (turquoise symbols), respectively. Mice with uninfected implants served as controls (white symbols). On day 10 after infection, animals were euthanized, edema sizes around the insertion site were determined, blood was taken, and the catheter fragments and the surrounding tissues (peri-implant tissues) were removed and separated. Bacteria adherent to the catheters were detached by sonication in saline, and tissue samples were homogenized in saline. Bacterial loads from catheter-detached biofilms (**a**) and in peri-implant tissue homogenates (**b**) were determined by CFU counting. Edema endpoints at day 10 post-infection are depicted in (**c**), and neutrophil numbers observed in the blood are depicted in (**d**). Each symbol represents an individual infection site, and horizontal bars indicate the median of all observations. *ns,* not significant; *p < 0.05; **p < 0.01 (Kruskal–Wallis test and Dunn’s multiple comparison test).

To elucidate whether the clear differences in biofilm formation between strains Newman HOM and Newman D2C HOM observed in our in vitro biofilm assays are also seen in the in vivo settings, we tested both strains in a murine foreign body-related infection model^64^. When fragments of PVC tubing were inserted into the flanks of normoglycemic C57BL/6 mice infected with strains Newman HOM and Newman D2C HOM, respectively, rather comparable bacterial numbers were isolated from the catheter tubing at day 10 post-infection (Fig. 9a).

However, both strains differed clearly in their capacities to multiply in the peri-implant tissue surrounding the catheter tubing fragment (Fig. 9b) and to cause inflammation at the insertion site (Fig. 9c), respectively. No bacteria were detected in blood samples taken from the animals at 10 days post infection, indicating that the infections of the catheter fragments and the peri-implant tissues did not become systemic. The latter hypothesis was also supported by neutrophil counts in blood, which were for both, Newman HOM and Newman D2C HOM-infected mice on a similar level as those observed in the blood of uninfected mice (Fig. 9d). Uninfected catheter fragments taken from the animals 10 days after implantation were all free of bacteria, and in only one mouse, lower numbers of bacteria were observed in the peri-implant tissues (Fig. 9a,b), suggesting that biofilms found on the catheter fragments at 10 days post-infection probably originated from the bacterial inocula. Overall, our findings made with the foreign body-related infection model are in line with earlier observations made with a murine sepsis model indicating that strain Newman forms larger bacterial loads in different organ tissues than strain Newman D2C^44^. Notably, our findings also fit with earlier observations indicating that substantial differences between staphylococcal wild type and isogenic mutant strains observed in microplate-based biofilm assays are not necessarily mirrored in the in vivo setting^65,66^. One reason for this might be the immune response of the host in the in vivo setting that is completely missing in the in vitro test systems. Another reason might be the availability of glucose in the test system, one of the preferred carbon sources of *S. aureus* for growth in culture media^67^. We demonstrated earlier that the capacity of *S. aureus* to form biofilms in a microplate-based assay relies largely on the amount of glucose that is available in the growth medium, and that only rather weak biofilms are formed when starting glucose concentrations in growth media such as TSB and brain heart infusion (BHI) are below 15 mM^62^. Notably, such high glucose levels are usually not observed in tissues or bodily fluids of normoglycemic mice^68,69^. Additionally, *S. aureus* is suggested to use distinct central metabolic pathways to adapt to the different carbon sources that are available at the site of infection^70^. In line with this, we failed to observe a clear impact of a *S. aureus ccpA* mutant (lacking a functional catabolite control protein A) on biofilm formation in our foreign body-related murine infection model in normoglycemic mice^66^, albeit of the fact that biofilm formation of *S. aureus* was greatly reduced by this mutation in a microplate-based biofilm assay^62^.

## Concluding remarks

We show here that our derivatives of the commonly used laboratory strains Newman and Newman D2C differ substantially in their capacities to adhere to and to form biofilms on abiotic surfaces in different in vitro assays. However, when tested in a murine foreign body-related murine infection model, both strains produced comparable vegetation on the implanted catheter tubing, although strain Newman HOM was more effective in multiplying in tissue surrounding the catheter tubing. The differences in biofilm formation observed for *S. aureus* Newman HOM and Newman D2C HOM in microplate-based in vitro assays, when compared to the results obtained with these strains in the murine foreign body-related infection model, call into question, how relevant findings made with the microplate-based in vitro assay are for the in vivo situation. Researchers working with strains Newman and Newman D2C should also be aware that both strains may differ substantially in their phenotypic behavior, and that isolate-to-isolate variation exist for a given strain in different laboratories, which might largely affect the outcome of the experiments. We would also like to point out that neither the strains nor the media, in vitro and in vivo assays presented in this study should be seen as best practice in *S. aureus* biofilm assays. For a critical discussion of these topics, we recommend reading the following reports/reviews^71–73^.

## Materials and methods

### Bacterial strains and sources

The bacterial strains used in this study and their relevant genotypes are listed in Table 2.

**Table 2:** *S. aureus* strains used in this study and their relevant genotypes.

Strain	Description*	References
Newman HOM	Strain isolated from a case of secondarily infected tubercular osteomyelitis, *saeS*	^29^
Newman D2C HOM	Clumping factor-positive variant of *S. aureus* strain Newman D2 (ATCC 25904), *agrA*, *saeR*	^28^
SA113	PIA-dependent biofilm producer, *agr*, *rsbU*	^25^

*PIA polysaccharide intercellular adhesin.

The *S. aureus* Newman derivative (hereinafter referred to as strain Newman HOM) originated from the Department of Microbiology at Trinity College Dublin, Ireland, and was transferred via the Universities of Geneva and Zurich, Switzerland, in 2007 to the Institute for Medical Microbiology and Hygiene (IMMH) at Saarland University, Homburg, Germany. The *S. aureus* Newman D2C derivative (hereinafter referred to as strain Newman D2C HOM) was obtained from the Institute for Medical Microbiology at Münster University, Germany, and transferred to IMMH in 2004. *S. aureus* strain SA113 was obtained in 2010 from the Interfaculty Institute of Microbiology and Infection Medicine at Tübingen University, Germany.

### Bacterial growth conditions

*S. aureus* strains were routinely grown on BD trypticase™ soy agar II with 5% sheep blood (SBA; BD 254087, BD, Heidelberg, Germany) at 37 °C, or as overnight cultures in BD Bacto™ tryptic soy broth (TSB; BD 211825, BD). Growth kinetics studies were carried out essentially as described in^74^. Briefly, bacterial cells from overnight cultures were diluted in TSB to an optical density at 600 nm (OD_600_) of 0.05, and bacterial cultures were incubated at 37 °C and 225 rpm with a flask-to-medium ratio of 10:1. Growth kinetics of cultures were monitored by taking samples at every hour and measuring the OD_600_ values using a Gene Quant 1300 spectrophotometer (Biochrom, Berlin, Germany). Cell suspensions reaching an OD_600_ > 0.8 were diluted with TSB and the measured absorbance values were multiplied by the dilution factor. The generation times of *S. aureus* strains cultivated in TSB under aerobic conditions were determined as described in^64^.

### Whole genome sequencing

For DNA preparation, bacteria were streaked out on SBA and incubated for 18 h at 37 °C. Grown colonies were scraped off the agar plate and transferred into a pathogen lysis tube provided by ZymoBIOMICS DNA Miniprep Kit (Zymo Research Crop., Irvine, USA) and mixed with the 750 µl of lysis buffer from the Kit. For mechanical and chemical lysis, the lysis tube containing bacterial cells and lysis buffer was placed into a MP Biomedicals FastPrep-24 5G bead beating lysis system (Fisher Scientific GmbH, Schwerte, Germany). For nucleic acid purification, we followed the manufacturer´s protocol and adjusted the velocity and duration of the mechanical lysis step to 6 m/s for 45 s. This step was repeated three times with 30 s of breaks in between and storage on ice. Nucleic acid isolation and purification were carried out following the manufacturer´s protocol, and bacterial DNA was eluted in 75 µl DNase-/RNase-free water. The concentration and purity of the samples was determined using the NanoDrop 2000/2000c full-spectrum microvolume UV/Vis measurement system (ThermoFisher Scientific, Karlsruhe, Germany).

For short-read sequences, library preparation and paired-end (PE150) Illumina sequencing (HiSeq) was done by Novogene Company Limited (Cambridge, UK) as described in^75^. For each sample, 3 gigabyte of short sequencing reads were achieved. For long-read sequences, library preparation and Nanopore sequencing were done as described in^76^, and for each isolate, about 2 gigabyte of long sequencing reads were produced.

### Sequencing data analysis

Quality control of short sequencing reads was performed with fastp (v:0.20) assessing also sequence overrepresentation^77^. Reads were aligned against the NZ_LT598688 reference genome using bwa-mem2 (v:2.2; samtools v:1.15)^78,79^. Variant calling of single nucleotide variants was performed with freebayes (v:1.3.2)^80^. Variants were filtered with vcflib (v:1.0.3; cla:*vcffilter -f 2*)^81^. The data analysis pipeline was organized with Snakemake (v:6.15.1)^82^. Initially tested short read assembly leveraged SPAdes (v:3.15.5; cla:*-isolate*)^83^. Long-read sequencing analysis relied on sniffles (v: 2.0.7)^84^ and minimap (v: 2.24 ; cla:*-ax map-ont*)^85^, respectively. The detected variant was manually inserted. Visualization of variants was realized with circilize in R^86^. Genetic alterations found between NZ_LT598688 and our Newman/Newman D2C genome assemblies were confirmed with EMBOSS Stretcher^87^. The genomic data are available at NCBI under the accession numbers CP160002.1 (Newman D2C HOM) and CP160003.1 (Newman HOM).

### RNA isolation and purification, cDNA synthesis and qRT-PCR

As described above, *S. aureus* strains were cultivated in TSB, and bacterial cells were sampled after 2 h and 6 h of incubation, respectively. Sample collection, disruption of bacteria, total RNA isolation, cDNA transcription, and qRT-PCRs were carried out as described in^88^ using the primers listed in Table 3. Transcriptional levels of target genes were normalized against the mRNA concentration of the housekeeping gene *gyrB* according to the 2^−ΔCT^ method.

**Table 3:** qRT-PCR primers used in this study.

Primer	Direction	Sequence (5′–3′)
*gyrB*	Forward	GACTGATGCCGATGTGGA
	Reverse	AACGGTGGCTGTGCAATA
*eap*	Forward	ATTTTATCAAGCTTAACATTTAATAAGAATCAA
	Reverse	ACTGATTTAACTCTATCCTCTAAATCTTTATAACTAAT
*RNAIII*	Forward	AGGAGTGATTTCAATGGCACAAG
	Reverse	TGTGTCGATAATCCATTTTACTAAGTCA

### Flow cytometry of 6 h TSB batch cultures

*S. aureus* strains were cultivated for 6 h in TSB as described above, and aliquots of the culture diluted 1:100 in PBS that has been passed through a 0.2 µm filter (Sarstedt, Nümbrecht, Germany) to remove larger particles. Diluted cell suspensions were supplemented with 3.34 µM of the fluorescent dye SYTO9 and/or 20 µM of propidium iodide (PI; Molecular Probes LIVE/DEAD kit L7012, ThermoFisher Scientific), respectively, and incubated for 10 min in the dark, before the stained cell suspensions were analyzed by fluorescence activated cell sorting using a Sony SH800 (Sony, Berlin, Germany). Megamix-Plus FCS beads (BioCytex, Marseille, France) and 3 µM FITC-labeled beads (Dako by Agilent, USA) were used for size vs. fluorescence calibration. Unstained cell suspensions and unstained/stained cell suspensions that were either heat treated (10 min at 80 °C) or sonicated (5 min at 50 W) served as controls for bacterial death or single particle formation, respectively. The gating strategy is indicated in Supplementary Fig. S1 online. Data were analyzed and visualized with FlowJo 10.6.2 software (Tree Star Inc., Ashland, OR, USA).

### Quantitative adhesion assay

Primary adhesion of *S. aureus* strains was tested with fluorescence-labeled exponential growth phase cells in Nunc™ MicroWell™ 96-Well, Nunclon Delta-treated, flat-bottom microplates (Fisher Scientific GmbH). Briefly, bacterial cells were cultured for 2.5 h in 5 ml of TSB as outlined above. 1 ml aliquots of the bacterial cell cultures were centrifuged by 12,000*g* for 1 min, and cell pellets were resuspended with 1 ml of PBS by pipetting the solution up and down. This procedure was redone twice before washed cells were resuspended in 500 µl of a phosphate buffered saline (PBS)-solution containing 10 µg/ml Invitrogen™ Hoechst 33342 (ThermoFisher Scientific). Cells were stained for 1 h at 37 °C and 500 rpm. Stained cells were centrifuged as outlined above, and the unbound dye was carefully removed by pipetting. The fluorescence-labeled cells were washed thrice with 1 ml PBS as outlined above, sonicated for 15 s at 50 W with an ultrasonic probe sonicator (Labsonic 1510, BBraun, Melsungen, Germany), and adjusted with PBS to an OD_600_ of 0.01 (using a GeneQuant™ 1300 spectrophotometer). 200 µl aliquots of this solution were pipetted into the wells of the 96-well microplate, and bacteria were allowed to adhere to the bottom of the wells for 1 h at 37 °C. After this incubation step, bacterial cultures were carefully removed, and adherent cells were washed 4 times with 200 µl of PBS by pipetting. Bacterial cells that remained on the bottom of the wells were covered with 100 µl PBS, and images of Hoechst 33342-labeled, adherent cells were taken with a Leica DMI 4000 B inverted fluorescence microscope equipped with a Leica DFC420 C camera and the Leica Application Suite software version V4.6.1 (Leica, Wetzlar, Germany). Images were acquired with a Leica N-Plan 20 × 0.40 numerical aperture objective using the DAPI filter setting. The number of adherent cells per 100 µm^2^ was determined manually on three images per well.

### Single-cell force spectroscopy (SCFS)

Both *S. aureus* strains were grown for SCFS as described above. After 2.5 h, the bacteria were washed thrice and then immobilized on the front end of a tipless cantilever (MLCT-O-F, Bruker-Nano, Santa Barbara, USA), following the protocol described by Thewes et al.^89^. Briefly, a thin layer of polydopamine was applied to the tipless cantilever by polymerizing dopamine hydrochloride (99%, Sigma-Aldrich, St. Louis, MI, USA) in Tris(hydroxymethyl)-aminomethane (TRIS) buffer (pH 7.4). Then, a single bacterium was attached to the polydopamine-coated tipless cantilever at the free end using a micromanipulator (Narishige Group, Tokyo, Japan). Care was taken to ensure that the cells did not dry out during probe preparation or force measurements. Before each measurement, the cantilever was calibrated using the Sader method^90^. Force spectroscopy measurements were performed under ambient conditions in PBS using a Nanowizard 4 (Bruker Nano GmbH, Berlin, Germany). All force-distance curves were obtained using parameters similar to prior studies^7,12,14,16^. The ramp size was set to 1600 nm, the force trigger (i.e., the maximum force with which the bacteria are pressed against the substrate) was 300 pN, and the retraction speed was 800 nm/s, with a surface time delay of 5 s. Prior research has shown a correlation between cell adhesion strength and cell-surface contact time^7,91–95^. Hence, a surface delay of 5 s was chosen.

Single, viable bacterial cells were subjected to force–distance measurements on a polystyrene surface (TPP Techno Plastic Products AG, Trasadingen, Switzerland) or PVC tubing (Vasofix^®^ Braunüle^®^ 1,30 × 45 mm 18 G; BBraun, Melsungen, Germany). Each bacterial probe and substrate were tested in a 10 μm by 10 μm grid, and 64 force-distance curves were recorded. The force-distance data was analyzed using a homemade MATLAB script^96^.

### Biofilm assays

Biofilm formation in microplate wells is described here following the minimum information guideline for spectrophotometric and fluorometric methods to assess biofilm formation in microplates^97^: Starting from three individual overnight cultures (classified as biological replicates), strains were cultured for 2.5 h in TSB as outlined above. The exponential growth phase cultures were diluted to an OD_600_ of 0.05 either in fresh TSB supplemented with 0.5% glucose (TSB-G; final glucose conc. 0.75%) or in TSB supplemented with 5% human blood serum (TSB-HBS; human off-the-clot serum, Bio&SELL GmbH, Feucht, Germany) or in Roswell Park Memorial Institute 1640 Medium (RPMI-1640; Gibco 11835-063, ThermoFisher Scientific). Two 150 µl aliquots (technical replicates) of these suspensions were transferred into the wells of Nunc™ MicroWell™ 96-Well, Nunclon Delta-treated, flat-bottom microplates and incubated for 18 h at 37 °C in a non-humidified incubator (Heraeus model B 5042 E, Heraeus, Hanau, Germany) under static conditions. For dynamic conditions, microplates were placed on an orbital shaker (Thermo Scientific™ model 88881102, 19 mm orbit; Fisher Scientific GmbH) at 120 rpm in a non-humidified incubator. At the end of the incubation period, growth media were carefully removed using a micropipette (Eppendorf Research Plus 20–200 µl, Eppendorf, Hamburg, Germany) fitted with a 200 µl tip (Sarstedt) inserted slowly at a 45° angle to the bottom of the well. Formed vegetation were washed twice with 200 µl of PBS using a micropipette fitted with a 200 µl tip. Remaining liquid residues were removed from the wells by tapping the plate three times on a stack of cellulose paper, and the plate was left upside down for 20 min in a biosafety cabinet (HeraSafe, Heraeus) under laminar flow at RT (21 ± 2 °C). Vegetation were stained with 150 µl of 0.1% (v/v) safranin (Merck, Darmstadt, Germany) for 30 s under static conditions at RT. As outlined above, the dye was removed by pipetting, stained wells were washed twice with 200 µl of distilled water, and the plate was left for 15 min upside down in a laminar flow. The stain was eluted with 200 µl of 30% (v/v) acetic acid (Merck) per well for 10 min at RT under static conditions. The eluted stain was mixed by pipetting up and down three times, and 150 µl/well of it was transferred to an empty 96-well optical-bottom plate (Thermo Scientific™ 96 well black/clear bottom plate, non-treated surface, no lid, non-Sterile; Fisher Scientific GmbH) using a micropipette. The absorbance was measured at 530 nm using a multimode microplate reader (Ensight, Perkin-Elmer, Rodgau, Germany). Means of technical replicates were corrected by subtracting the corresponding *A*_530_ readings of the negative control (TSB only). To determine the impact of washing on biofilm removal, wells were washed either twice, once, or left without washing before remaining medium/PBS residues were removed from the wells by tapping them out.

To quantify PIA contents in the biofilms formed by the *S. aureus* strains, we followed a labeling protocol described by Skogman et al.^98^. Exponential growth phase cells were cultured in TSB-G in 96-well plates under static conditions as described above. After 18 h of growth, wells were washed once with PBS and vegetation covered with 150 µl of a PBS solution supplemented with 2.5 µg/ml (w/v) XFD488-conjugated wheat germ agglutinin (XFD488-WGA; AAT Bioquest, Biomol GmbH, Hamburg, Germany) that binds to PIA and allows visualization of PIA localization. Vegetation were incubated for 2 h at 4 °C in the dark before the labeling solution was removed by pipetting as outlined above. Wells were washed twice with 200 µl of PBS, and the remaining liquid residues were removed by tapping the plate onto cellulose paper and labeled vegetation air-dried for 15 min in a laminar flow. For the elution of the bound probe, wells were filled with 200 µl of 30% (v/v) acetic acid per well and filled wells covered with strip caps (Nunc™ 8-well strip cap, Fisher Scientific GmbH). Microplates were sonicated twice in an ultrasonic bath (BactoSonic BS 14.2, Bandelin electronic GmbH, Berlin, Germany) for 30 s, interrupted by an incubation step of 1 h at 37 °C in the dark. The eluted probes were mixed by pipetting up and down three times, and 150 µl aliquots per well were transferred to an empty 96-well optical-bottom plate using a micropipette. The fluorescence of the samples was read from the top of the plate at an excitation wavelength of 480 nm and an emission wavelength of 530 nm using an EnSight multimode microplate reader (Perkin-Elmer).

For the visualization of the three-dimensional structures of the biofilms formed by *S. aureus* in microplates, exponential growth phase cells were diluted in TSB-G to an OD_600_ of 0.05, and 1 ml aliquots placed into the wells of a sterile flat bottom, tissue culture-treated six-well microplate (TPP 6-well tissue culture plate; Merck). The inoculated plate was cultured for 18 h at 37°C under static conditions as outlined above. For staining of PIA, wells were washed once with PBS, and vegetation were covered with 1 ml of a PBS solution supplemented with 2.5 µg/ml (w/v) XFD488-WGA (AAT Bioquest). Vegetation were incubated for 2 h at 4°C in the dark before the labeling solution was removed by pipetting using a micropipette (Eppendorf Research Plus 100–1000 µl) fitted with a 1000 µl tip (Sarstedt) as outlined above. For fluorescence labeling of the bacterial cells, vegetation were subsequently covered with 1 ml of a PBS solution supplemented with 10 µg/ml (w/v) of the lipophilic stain Nile Red (NR; Invitrogen No. N1142, ThermoFisher Scientific) and incubated for 15 min at RT. After staining, the NR solution was removed by pipetting as outlined above, and stained biofilms were covered with 1 ml of PBS. Confocal laser scanning microscopy (CLSM) was performed with a Nikon A1R upright confocal microscope system (Nikon; Düsseldorf, Germany) and NIS Elements software (NIS Elements AR 3.2, 64 bit; Nikon). Images were acquired with a 10 × 0.30 numerical aperture water objective. Standard filter sets were used to capture XFD488-WGA and NR emissions, with excitation at 488 nm and 561 nm, respectively. For qualitative analyses, confocal images from all three *S. aureus* strains were acquired under identical conditions by using the ‘’re-use camera settings’’ option of the NIS elements software. To generate 3D images, the Nikon RFA Z-drive plugin was connected to the NIS Elements software. Z-stacks were recorded with an interval of 20 μm to scan the entire thickness or width of the biofilm, with a calibration of 2.5 μm/px. For each sample, 51 optical slices were obtained and used for 3D reconstruction with the NIS Elements software (Alpha density viewing function).

For the assessment of biofilm formation on medical devices under dynamic conditions, the following two assays were used: In assay one, PVC (Venflon Pro Safety 18 G; BD) fragments of 1 cm length were placed into 2 ml reaction tubes and the lumen of the catheter fragments inoculated with 10 µl of exponential growth phase TSB cultures (diluted to an OD_600_ of 0.5 in fresh TSB) of strains SA113, Newman HOM and Newman D2C HOM, respectively. Reaction tubes were filled with 1.8 mL of TSB-G and RPMI-1640, respectively, and the catheter tubing fragments incubated for five days at 37 °C and 20 rpm in a rotating mixer (Stuart rotator SB3, VWR, Darmstadt, Germany). Media were replaced with fresh media every 24 h. At the end of the incubation time, PVC fragments were taken out of the reaction tube with a sterile 200 µl tip, and the liquid in the lumen removed by gently passing air through the lumen. The vegetated tubing fragments were next placed into fresh reaction tubes filled with 1 ml of TSB, and biofilms were detached from the catheter surface and resolved by sonication (50 W for 5 min) followed by 1 min of vortexing. CFU rates of resolved biofilms at day five post inoculation were determined by plate counting.

To test for the biofilm formation capacities of the strain set under flow conditions, the following set-up was used: The bacterial inoculum was prepared as outlined above, and a TSB culture with an OD_600_ of 0.1 were used to infect the tubing of the PVC (Venflon Pro Safety 18 G; BD) by placing the tip of the tube into the bacterial solution and filling half of the tube with the bacterial solution by drawing up a syringe (Inject Luer Solo 5 ml, B. Braun). Cells were allowed to attach to the inner walls of the channel for 90 min at 37 °C under static conditions. At the end of the incubation time, the syringe was removed, the inlet of the PVC connected via tubes and a peristaltic pump (Ismatec REGLO Digital; Postnova, Landsberg am Lech, Germany) to a pre-warmed medium reservoir filled with sterile TSB, and the outlet of the PVC placed above a waste reservoir. Afterwards, medium was pumped through the PVC at a flow rate of 1 ml per min for 18 h. At the end of the experiment, the inoculated part of the tubing was cut off with scissors, the length of the tubing determined, and the tubing cut into 2 pieces. Tubing pieces were placed into a sterile 2 ml reaction tube filled with 1 ml of fresh TSB, and biofilm detachment and bacterial load determinations were carried out as described above.

### Murine infection model

Animal experiments were performed with the approval of the local State Review Board of Saarland, Germany (project identification code 14/2021) and were conducted following the national and European guidelines for the ethical and human treatment of animals. All authors complied with the ARRIVE guidelines. The abilities of strains Newman HOM and Newman D2C HOM to form biofilms on implanted material under in vivo conditions was tested with a murine foreign body-related infection model, as described in^64^. Briefly, 8–10 week-old female C57BL/6 N mice (Charles River, Sulzfeld, Germany) were narcotized by intraperitoneal injection of fentanyl (0.05 mg per kg body weight [BW]; Hexal, Holzkirchen, Germany), midazolam (5 mg/kg BW; Hameln Pharma Plus, Hameln, Germany) and medetomidine (0.5 mg/kg BW; Orion Pharma, Hamburg, Germany). After treatment with a dose of carprofen (5 mg/kg BW; Zoetis, Berlin, Germany), the flanks of animals were shaved with an animal trimmer (BBraun, Melsungen, Germany) and subsequently depilated with Asid-med hair removal cream (Asid Bonz, Herrenberg, Germany). Depilated skin regions were disinfected with ethanol (70%) and the mouse placed in prone position. Using a sterile scissors (Fine Science Tools, Heidelberg, Germany), 5 mm long incisions were made in the cutis of the depilated skin regions. For each site, the cutis was carefully lifted away from the subcutis by spreading the scissors and a sterile 1 cm catheter tubing fragment (Venflon Pro Safety 18 G; BD) was placed in the gap between the cutis and subcutis so that one end of the fragment was still visible. For the inoculum, strains were cultured for 2.5 h in TSB as outlined above. The exponential growth phase cultures were centrifuged by 5.000 g for 5 min, the cell pellets washed twice with PBS and resuspended in fresh PBS to an OD_600_ of 0.5. The bacterial solution was diluted 1:100 in PBS, and 20 µl of this solution (~ 1 × 10^4^ CFU) was injected into the lumen of the PVC tubing using a micropipette fitted with a 200 µl tip. The catheter fragment was subsequently pushed slightly deeper into the gap, and wounds were closed with staples (Fine Science Tools). Afterwards, anesthesia was antagonized with naloxone (1.2 mg/kg BW; Inresa, Freiburg im Breisgau, Germany), flumazenil (0.5 mg/kg BW; Inresa) and atipamezole (2.5 mg/kg BW; Orion Pharma). Mice with PVC fragments implanted into the flanks that were not infected with *S. aureus* served as uninfected controls. Behavior and weight of the animals was monitored daily. At 10 days post infection, animals were anesthetized by intraperitoneal injection of ketamine hydrochloride (100 mg/kg BW; bela-pharm, Vechta, Germany) and xylazine hydrochloride (12 mg/kg BW; bela-pharm) and edema sizes were measured with a digital caliper (ChiliTec 17909, ChiliTec GmbH, Lehre-Essenrode, Germany). Animals were afterwards euthanized by injecting a lethal dose of ketamine hydrochloride (250 mg/kg BW) and xylazine hydrochloride (25 mg/kg BW). After the absence of the inter-phalangeal reflex, the animal’s chest was opened and blood was taken by cardiac puncture. Catheter tubing and peri-implant tissue that has formed around the implant were harvested. The animal was then bled by severing the aorta. Excised tissues were homogenized in 1 ml TSB with a POLYTRON PT 1200 E disperser (Kinematica, Eschbach, Germany). Biofilms formed on the PVC tubing were detached and resolved by sonification (50 W for 5 min) followed by vortexing (1 min). Bacterial loads in tissue and on the catheter were determined by plating serial dilutions on sheep blood agar plates and plate counting after 24 h of incubation at 37 °C. A blood stripe incubated on SBA at 37 °C served as an indicator of systemic bacterial infection. Leukocytes in the blood of infected mice at day 10 post-infection were determined as described in^99^.

### Statistical analyses

The statistical significance of changes between groups was assessed by the Mann–Whitney *U* test (direct comparisons of two groups) or the Kruskal–Wallis test followed by Dunn’s multiple comparison test (three groups) using the GraphPad software package Prism 9.5.1 (San Diego, CA 92108, USA). *p* values < 0.05 were considered statistically significant.

## Electronic supplementary material

Below is the link to the electronic supplementary material.

Supplementary Material 1

## Data Availability

Next generation sequencing data generated in this study have been deposited in the Sequence Read Archive under the accession code PRJNA1124351. Further datasets generated during and/or analyzed during the current study are available from the corresponding author on reasonable request.
