# Supplementary material for: Assessment of the biofilm formation capacities of Staphylococcus aureus strains Newman and Newman D2C in vitro and in vivo
Source: Sci Rep. 2025 May 8;15:16132. doi: 10.1038/s41598-025-00521-5 (PMC12062259; doi:10.1038/s41598-025-00521-5)
Supplement: Supplementary file 1 — Supplementary Material 1 [file 41598_2025_521_MOESM1_ESM.pdf]

Assessment of the biofilm formation capacities of *Staphylococcus aureus* strains Newman and Newman D2C *in vitro* and *in vivo*

Ben Wieland[1], Gubesh Gunaratnam[1], Linda Pätzold[1], Noran Abdel Wadood[1,2], Georges Pierre Schmartz[3], Swarnali Kundu[1,2], Nikolay Krasimirov Kirilov[1], Ina Krüger[1], Mohamed Ibrahem Elhawy[1,2], Jacqueline Rehner[1], Hannah Heintz[4], Frank Schmitz[2], Daniela Yildiz[5], Gabriela Krasteva-Christ[2], Sören L. Becker[1], Karin Jacobs[4,6], and Markus Bischoff[1,*]

[1] Institute of Medical Microbiology and Hygiene, Saarland University, 66421 Homburg, Germany

[2] Institute of Anatomy and Cell Biology, Saarland University, 66421 Homburg, Germany

[3] Clinical Bioinformatics, Saarland University, 66123 Saarbrücken, Germany

[4] Experimental Physics, Center for Biophysics, Saarland University, 66123, Saarbrücken, Germany

[5] Preclinical Center for Molecular Signaling, Molecular Pharmacology, Saarland University, 66421 Homburg, Germany

[6] Max Planck School Matter to Life, 69120 Heidelberg, Germany

[*] Corresponding author

Supplementary Figures:

Figures S1-S2

Supplementary Tables:

Table S1

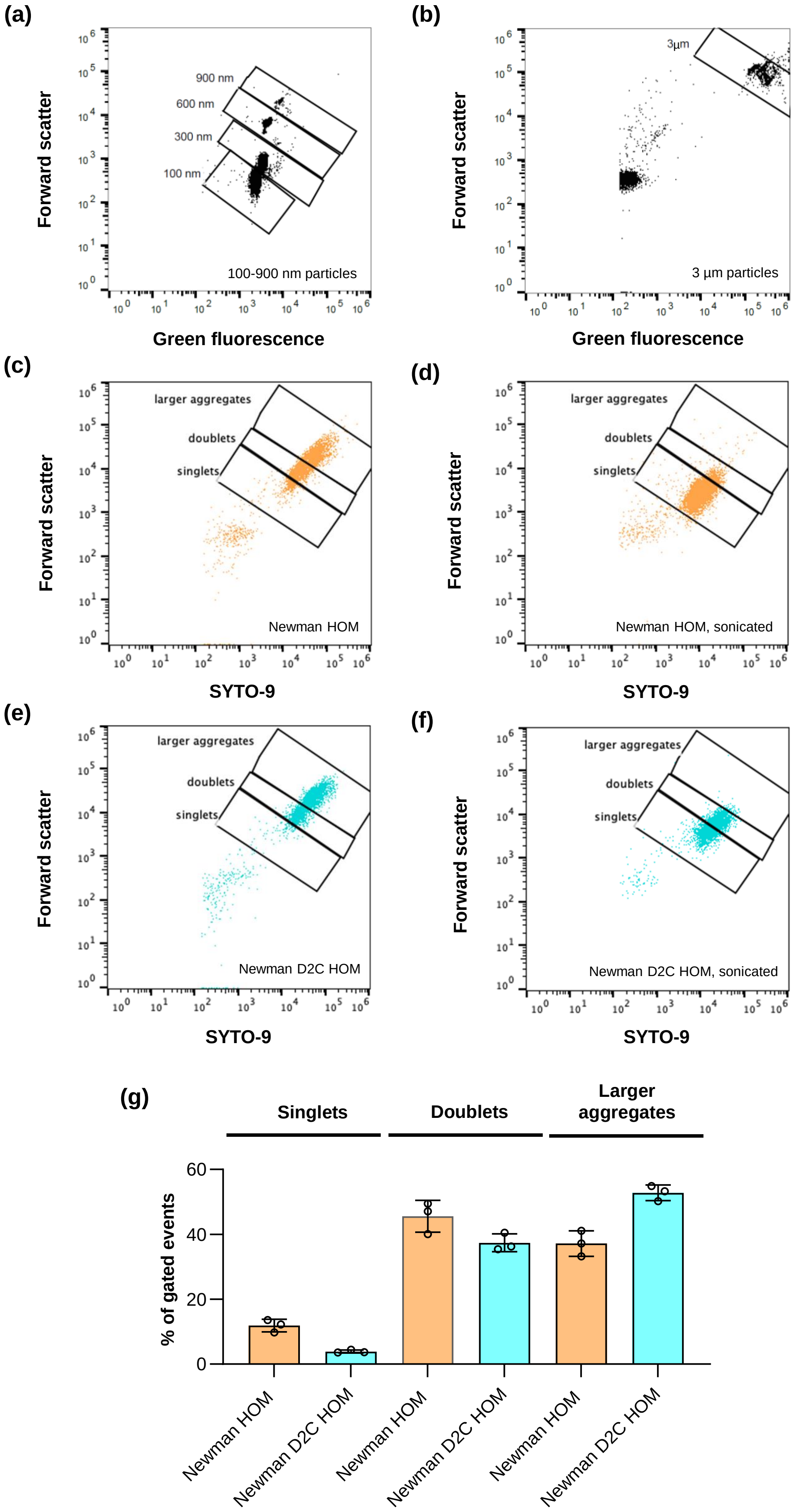

Figure S1: Gating strategy for the cell aggregate determination of Newman HOM and Newman D2C HOM cultures grown for 6 h at 225 rpm and 37°C in TSB. **(a-f)** Fluorescently labeled beads and SYTO-9 stained cells were detected by excitation at 488 nm (blue laser) and detected in fluorescence channel 1 (FL1, 525/50 nm). **(a, b)** Forward scatter vs. green fluorescence cytogram of Megamix-Plus FCS beads (a; 100, 300, 600, and 900 nm beads) and FITC-labeld 3 µm beads (b). Gates were transferred to samples of SYTO-9 stained bacteria (c-f). The single bacterial particle gate was identified after sonication of the bacterial suspensions (d, f), assuming an average size of 600 nm per bacterial particle (singlets). The resolution allowed for the separation of aggregates into doublets and larger aggregates. **(g)** Singlets, doublets and larger aggregates were quantified as percentage of events in the respective gates. Results represent the averages of three independent experiments. Error bars indicate the standard deviation of the mean. Round symbols indicate the values of individual experiments.

(a)

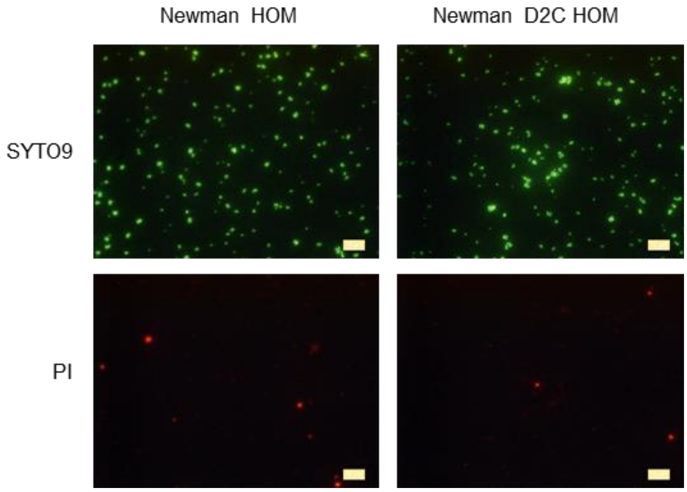

(b)

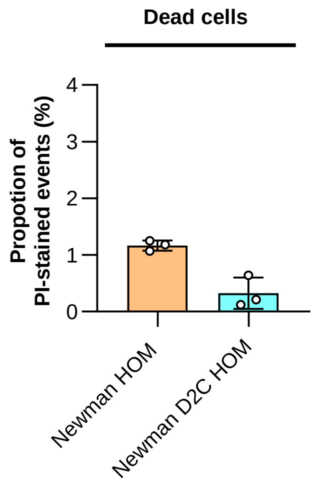

Figure S2: Proportion of dead cells in Newman HOM and Newman D2C HOM 6 h TSB cultures. **(a)** Representative micrographs of SYTO9/propidium iodide (PI)-stained *S. aureus* Newman HOM and Newman D2C HOM cultures. Strains were cultured in TSB for 6 h at 37°C and 225 rpm. Images of SYTO9/PI-stained cells were taken with a Leica DMI 4000 B inverted fluorescence microscope equipped with a Leica DFC420 C camera and the Leica application suite software version V4.6.1 (Leica, Wetzlar, Germany). Images were acquired with a Leica N-Plan 40 x 0.55 numerical aperture objective using the FITC and PI filter settings (Leica filter cubes I3 and N2.1, respectively). **(b)** Proportion of PI-stained events. Cell suspensions were stained and FACS-analyzed as outlined in Fig. S1. SYTO-9 stained cells were detected by excitation at 488 nm (blue laser) and detected in fluorescence channel 1 (FL1, 525/50 nm). PI-labeled bacteria were detected by excitation at 561 nm (yellow-green laser) and detected in fluorescence channel 3 (FL3, 617/30 nm). Proportions of PI-positive cells were determined in relation to the numbers of SYTO-9 stained cells, which were set as 100%. Results represent the averages of three independent experiments. Error bars indicate the standard deviation of the mean. Round symbols indicate the values of individual experiments.

Table S1: List of nucleotide polymorphisms identified in the genomes of strains Newman HOM (CP160003.1), Newman (AP009351.1), NYU Newman (CP023390.1), Newman D2C NY (CP023391.1), and Newman D2C HOM (CP160002.1) in relation to the Newman UoM reference genome (NZ_LT598688.1)

Genomic position NZ_LT598688.1	REF	ALT	Mutation type	Product name	Gene	Strand	Nucleotide change	Protein change	Consequence	Newman UoM ref. Genome (NZ_LT598688.1)	Newman HOM (CP160003.1)	Newman (AP009351.1)	NYU Newman (CP023390.1)	Newman D2C NY (CP023391.1)	Newman D2C HOM (CP160002.1)
2047/8	CA	C**CA**A	ins	intergenic region	-	-	NA	NA	potential change in transcription of dnaN			1	1	1	
2068/9	TA	T**TA**A	ins	intergenic region	-	-	NA	NA	potential change in transcription of dnaN			1	1	1	
2070/1	TC	T**TC**C	ins	intergenic region	-	-	NA	NA	potential change in transcription of dnaN			1	1	1	
2073	A	C	snp	intergenic region	-	-	NA	NA	potential change in transcription of dnaN			1			
2075	C	C**C**	ins	intergenic region	-	-	NA	NA	potential change in transcription of dnaN			1	1	1	
87710	G	A	snp	3-(L-alanin-3-ylcarbamoyl)-2-[(2-aminoethylcarbamoyl)methyl]-2-hydroxypropanoate synthase SbnF	sbnF	p	G1347A	no	none			1	1		
114106	G	A	snp	pseudogene	BN8422_RS15625	p	NA	NA	none		1				
114117/20	TTTT	TACT	mnp	pseudogene	BN8422_RS15625	p	NA	NA	none		1				
114126/31	CAAACT	CTAAAT	mnp	pseudogene	BN8422_RS15625	p	NA	NA	none		1				
114147/50	TTAA	TACA	mnp	pseudogene	BN8422_RS15625	p	NA	NA	none		1				
114165	T	C	snp	pseudogene	BN8422_RS15625	p	NA	NA	none		1				
114179	A	G	snp	pseudogene	BN8422_RS15625	p	NA	NA	none		1				
114186/94	AACTCTTT	AGATACGT	mnp	pseudogene	BN8422_RS15625	p	NA	NA	none		1				
114197	A	G	snp	pseudogene	BN8422_RS15625	p	NA	NA	none		1				
114207	G	A	snp	pseudogene	BN8422_RS15625	p	NA	NA	none		1				
114214	C	T	snp	pseudogene	BN8422_RS15625	p	NA	NA	none		1				
114229	T	A	snp	pseudogene	BN8422_RS15625	p	NA	NA	none		1				
114235	G	A	snp	pseudogene	BN8422_RS15625	p	NA	NA	none		1				
114239	T	A	snp	pseudogene	BN8422_RS15625	p	NA	NA	none		1				
114247	T	A	snp	pseudogene	BN8422_RS15625	p	NA	NA	none		1				
114251	G	A	snp	pseudogene	BN8422_RS15625	p	NA	NA	none		1				
114270/4	ATATCA	ACATTA	mnp	pseudogene	BN8422_RS15625	p	NA	NA	none		1				
114276/84	CAGTTATTT	CGACTACAT	mnp	pseudogene	BN8422_RS15625	p	NA	NA	none		1				
114287/96	ACGATGTTAG	AAGACGATGG	mnp	pseudogene	BN8422_RS15625	p	NA	NA	none		1				
114304	G	A	snp	pseudogene	BN8422_RS15625	p	NA	NA	none		1				
114316	G	A	snp	pseudogene	BN8422_RS15625	p	NA	NA	none		1				
114333	T	A	snp	pseudogene	BN8422_RS15625	p	NA	NA	none		1				
114342	A	G	snp	pseudogene	BN8422_RS15625	p	NA	NA	none		1				
114346	C	T	snp	pseudogene	BN8422_RS15625	p	NA	NA	none		1				
114355/62	ATTGTCAG	AATGACTG	mnp	pseudogene	BN8422_RS15625	p	NA	NA	none		1				
114366/74	AGTCGGTAA	AATCAGTTA	mnp	pseudogene	BN8422_RS15625	p	NA	NA	none		1				
114385	A	T	snp	pseudogene	BN8422_RS15625	p	NA	NA	none		1				
114394	A	T	snp	pseudogene	BN8422_RS15625	p	NA	NA	none		1				
114399/404	ATGTAA	ACGGTA	mnp	pseudogene	BN8422_RS15625	p	NA	NA	none		1				
114415	A	G	snp	pseudogene	BN8422_RS15625	p	NA	NA	none		1				
114430	G	T	snp	pseudogene	BN8422_RS15625	p	NA	NA	none		1				
114433/7	TAATA	TGACA	mnp	pseudogene	BN8422_RS15625	p	NA	NA	none		1				
114445	G	A	snp	pseudogene	BN8422_RS15625	p	NA	NA	none		1				
114451/5	TCGGA	TAGAA	mnp	pseudogene	BN8422_RS15625	p	NA	NA	none		1				
114468/72	A-CAAG	A**A**CAGG	mnp	pseudogene	BN8422_RS15625	p	NA	NA	none		1				
114477	T	C	snp	pseudogene	BN8422_RS15625	p	NA	NA	none		1				
114492/7	TGTTGA	TATTAA	mnp	pseudogene	BN8422_RS15625	p	NA	NA	none		1				
114501/7	TGATTC--A	TATGTC**TG**A	mnp	pseudogene	BN8422_RS15625	p	NA	NA	none		1				
114510/5	ATT**TC**T	ACT--T	mnp	pseudogene	BN8422_RS15625	p	NA	NA	none		1				
114525	G	A	snp	pseudogene	BN8422_RS15625	p	NA	NA	none		1				
114541	C	A	snp	pseudogene	BN8422_RS15625	p	NA	NA	none		1				
114557	G	A	snp	pseudogene	BN8422_RS15625	p	NA	NA	none		1				
114564	A	G	snp	pseudogene	BN8422_RS15625	p	NA	NA	none		1				
114574	G	A	snp	pseudogene	BN8422_RS15625	p	NA	NA	none		1				
114582	G	A	snp	pseudogene	BN8422_RS15625	p	NA	NA	none		1				
114663	G	A	snp	pseudogene	BN8422_RS15625	p	NA	NA	none		1				
114675	T	A	snp	pseudogene	BN8422_RS15625	p	NA	NA	none		1				
114693	A	G	snp	pseudogene	BN8422_RS15625	p	NA	NA	none		1				
214713	G	A	snp	thiolase family protein	BN8422_RS00925	m	C687T	no	none						1
267700	C	T	snp	intergenic region	-	-	NA	NA	potential change in transcription of LytM			1	1	1	
355667	G	T	snp	phage baseplate upper protein	BN8422_RS01740	p	G646T	V220F	potential change in activity of BN8422_RS01740		1				1
355813	T	C	snp	phage baseplate upper protein	BN8422_RS01740	p	T792C	no	none		1				1
355821	A	C	snp	phage baseplate upper protein	BN8422_RS01740	p	A800C	Y267S	potential change in activity of BN8422_RS01740		1				1
355850	G	A	snp	phage baseplate upper protein	BN8422_RS01740	p	G829A	A277T	potential change in activity of BN8422_RS01740		1				1
356635	A	T	snp	phage baseplate upper protein	BN8422_RS01740	p	A1614T	no	none		1				1
359149/56	GGCAAGAG	GTCTAGGG	mnp	glucosaminidase domain-containing protein	BN8422_RS01760	p	G1276T, A1278T, A1281G	A426S	potential change in activity of BN8422_RS01760		1				1
359173/80	TAATGCTT	TCATACAT	mnp	glucosaminidase domain-containing protein	BN8422_RS01760	p	A1300C, G1303A, T1305A	N434H, A435T	potential change in activity of BN8422_RS01760		1				1
359186	T	C	snp	glucosaminidase domain-containing protein	BN8422_RS01760	p	T1311C	no	none		1				1
359193/201	ACGCTA	ATGCAA	mnp	glucosaminidase domain-containing protein	BN8422_RS01760	p	C1320T, T1323A	no	none		1				1
359214/9	GTTCAG	GCTCGG	mnp	glucosaminidase domain-containing protein	BN8422_RS01760	p	T1341C, T1344A	no	none		1				1

359233	A	G	snp	glucosaminidase domain-containing protein	BN8422_RS01760	p	A1359G	no	none		1				1
359257	G	A	snp	glucosaminidase domain-containing protein	BN8422_RS01760	p	G1383A	no	none		1				1
359263	C	T	snp	glucosaminidase domain-containing protein	BN8422_RS01760	p	C1389T	no	none		1				1
359294	G	A	snp	glucosaminidase domain-containing protein	BN8422_RS01760	p	G1420A	V474I	potential change in activity of BN8422_RS01760		1				1
359306	T	G	snp	glucosaminidase domain-containing protein	BN8422_RS01760	p	T1432G	S478A	potential change in activity of BN8422_RS01760		1				1
359313/8	ATAAGA	ACAAAA	mnp	glucosaminidase domain-containing protein	BN8422_RS01760	p	T1440C, G1443A	no	none		1				1
359376/81	AAAAGCA	AGAAAAA	mnp	glucosaminidase domain-containing protein	BN8422_RS01760	p	A1503G, G1506A, C1507A	H503N	potential change in activity of BN8422_RS01760						1
359392	T	C	snp	glucosaminidase domain-containing protein	BN8422_RS01760	p	T1518C	no	none		1				1
359404	A	G	snp	glucosaminidase domain-containing protein	BN8422_RS01760	p	A1530G	no	none		1				1
359413	T	C	snp	glucosaminidase domain-containing protein	BN8422_RS01760	p	T1539C	no	none		1				1
359434	T	C	snp	glucosaminidase domain-containing protein	BN8422_RS01760	p	T1560C	no	none		1				1
371815	C	A	snp	aldehyde reductase	BN8422_RS01825	p	C681A	D227E	potential change in activity of BN8422_RS01825		1				
392066/72	TAAACAA	TTTAACA	mnp	intergenic region	-	-	NA	no	potential change in transcription of BN8422_RS01940			1	1	1	
394457	A	C	snp	low temperature requirement protein A	BN8422_RS01950	m	T602G	L201R	potential change in activity of BN8422_RS01950					1	1
423561	T	A	snp	hypothetical protein	BN8422_RS02120	m	A653T	K219N	potential change in activity of BN8422_RS02120			1	1	1	
456623/7	ATCAA	AATCA	mnp	tandem-type lipoprotein Lpl5	lpl5	p	T113A, C114T, A115C	I39N, K40Q	potential change in activity of Lpl5			1	1	1	
472768	G	A	snp	phosphatase PAP2 family protein	BN8422_RS02375	p	G189A	no	none			1	1	1	
472939	A	T	snp	phosphatase PAP2 family protein	BN8422_RS02375	p	A361T	no	none			1	1	1	
472993	G	A	snp	phosphatase PAP2 family protein	BN8422_RS02375	p	G415A	no	none			1	1	1	
473032	C	T	snp	phosphatase PAP2 family protein	BN8422_RS02375	p	C453T	no	none			1	1	1	
473044	T	A	snp	phosphatase PAP2 family protein	BN8422_RS02375	p	T465A	no	none			1	1	1	
473090	T	C	snp	phosphatase PAP2 family protein	BN8422_RS02375	p	T511C	no	none			1	1	1	
473092	G	A	snp	phosphatase PAP2 family protein	BN8422_RS02375	p	G513A	no	none			1	1	1	
473146	A	G	snp	phosphatase PAP2 family protein	BN8422_RS02375	p	A567G	no	none			1	1	1	
478391	G	A	snp	methionine ABC transporter ATP-binding protein	BN8422_RS02405	p	G23A	S8N	potential change in activity of BN8422_RS02405			1	1	1	
478393	A	T	snp	methionine ABC transporter ATP-binding protein	BN8422_RS02405	p	A25T	K9*	premature stop			1	1	1	
478398	C	T	snp	methionine ABC transporter ATP-binding protein	BN8422_RS02405	p	C30T	no	none			1	1	1	
478418/9	A**A**	A	del	methionine ABC transporter ATP-binding protein	BN8422_RS02405	p	A51-	no	frameshift mutation			1	1	1	
502644	T	G	snp	rRNA-16S ribosomal RNA	BN8422_RS02520	p	T165G	NA	unknown		1				1
504076	C	T	snp	intergenic region	-	-	NA	NA	none		1				1
504150/17	G**AAAATAAAGCAGTATG**C	GC	del	intergenic region	-	-	NA	NA	none		1				1
519329	G	T	snp	16S rRNA (adenine(1518)-N(6)/adenine(1519)-N(6))-dimethyltransferase RsmA	rsmA	p	G183T	M61I	potential change in activity of RsmA			1	1	1	
545819	A	G	snp	intergenic region	-	-	NA	NA	none					1	1
582024	G	A	snp	DNA-directed RNA polymerase subunit beta'	rpoC	p	G2239A	A747T	potential change in activity of RpoC				1		
586896	T	C	snp	504elongation factor G	fusA	p	T1954C	F652L	potential change in activity of FusA			1	1	1	
586923	A	C	snp	elongation factor G	fusA	p	A1981C	N661H	potential change in activity of FusA			1	1	1	
586974	G	A	snp	elongation factor G	fusA	p	G2032A	V678I	potential change in activity of FusA			1	1	1	
587073	T	C	snp	intergenic region	-	-	NA	no	none			1	1	1	
587108	T	C	snp	intergenic region	-	-	NA	no	none			1	1	1	
588666	G	T	snp	intergenic region	-	-	NA	no	none			1	1	1	
592106	C	A	snp	glyoxalase III HchA	hchA	p	C601A	L201I	potential change in activity of HchA			1	1	1	
618686	T	C	snp	glucosamine-6-phosphate deaminase	nagB	p	T279C	no	none					1	1
685403/5	AC**A**	A	del	M50 family metallopeptidase	BN8422_RS03445	m	T558-, G559-	multiple	truncated protein			1	1	1	
747605	G	A	snp	intergenic region	-	-	NA	NA	potential change in transcription of BN8422_RS03775					1	1
757763	G	A	snp	response regulator transcription factor SaeR	saeR	m	C595T	R199C	change in activity of SaeR					1	1
812216	C	A	snp	intergenic region	-	-	NA	NA	potential change in transcription of BN8422_RS04100					1	1
814755	A	G	snp	excinuclease ABC subunit UvrB	uvrB	p	A325G	T109A	potential change in activity of UvrB			1	1	1	
814763	T	G	snp	excinuclease ABC subunit UvrB	uvrB	p	T333G	I111M	potential change in activity of UvrB			1	1	1	
814800	C	G	snp	excinuclease ABC subunit UvrB	uvrB	p	C370G	L124V	potential change in activity of UvrB			1	1	1	
897816/7	A**A**	A	del	iron-sulfur cluster assembly accessory protein	BN8422_RS04590	p	NA	NA	ORF not annotated in AP009351.1			1	1	1	
897843	C	A	snp	iron-sulfur cluster assembly accessory protein	BN8422_RS04591	p	NA	NA	ORF not annotated in AP009351.1			1	1	1	
897844/5	A**G**	A	del	iron-sulfur cluster assembly accessory protein	BN8422_RS04592	p	NA	NA	ORF not annotated in AP009351.1			1	1	1	
897869/73	AA**TG**G	ATG	del	iron-sulfur cluster assembly accessory protein	BN8422_RS04593	p	NA	NA	ORF not annotated in AP009351.1			1	1	1	
897880	G	G**G**	ins	iron-sulfur cluster assembly accessory protein	BN8422_RS04594	p	NA	NA	ORF not annotated in AP009351.1			1	1	1	
897891	T	A	snp	iron-sulfur cluster assembly accessory protein	BN8422_RS04595	p	NA	NA	ORF not annotated in AP009351.1			1	1	1	
897894/5	C**A**	C	del	iron-sulfur cluster assembly accessory protein	BN8422_RS04596	p	NA	NA	ORF not annotated in AP009351.1			1	1	1	
897912/5	AAGA	AGAA	mnp	iron-sulfur cluster assembly accessory protein	BN8422_RS04597	p	NA	NA	ORF not annotated in AP009351.1			1	1	1	
897923/4	G**G**	G	del	iron-sulfur cluster assembly accessory protein	BN8422_RS04598	p	NA	NA	ORF not annotated in AP009351.1			1	1	1	
897953/4	G**G**	G	del	iron-sulfur cluster assembly accessory protein	BN8422_RS04598	p	NA	NA	ORF not annotated in AP009351.1			1	1	1	
897992/3	T**T**	T	del	iron-sulfur cluster assembly accessory protein	BN8422_RS04598	p	NA	NA	ORF not annotated in AP009351.1			1	1	1	
901400	A	A**A**	ins	intergenic region	-	-	NA	NA	Insertion immediatly downstream of BN8422_RS04600			1	1	1	
935736	G	T	snp	Cof-type HAD-IIB family hydrolase	BN8422_RS04750	m	C701A	A234D	potential change in activity of BN8422_RS04750					1	
936394	A	A**A**	ins	Cof-type HAD-IIB family hydrolase	BN8422_RS04750	m	NA	NA	frameshift mutation after L15						1

972712	G	A	snp	RluA family pseudouridine synthase	BN8422_RS04925	p	G288A	no	none			1	1	1	
996710/3	ATAA	AATA	mnp	IDEAL domain-containing protein	BN8422_RS05025	p	T217A, A218T	*72I	Elongated protein (74 AA) in AP009351.1			1	1	1	
996729	C	C**C**	ins	intergenic region	-	-	NA	NA	Potential change in transcriptional termination of BN8422_RS05025			1	1	1	
996737/8	AA	A	del	intergenic region	-	-	NA	NA	Potential change in transcriptional termination of BN8422_RS05025			1	1	1	
997911	T	G	snp	intergenic region	-	-	NA	NA	potential change in transcription of BN8422_RS05035			1	1	1	
1005331	C	G	snp	glycosyltransferase	BN8422_RS05080	p	C1341G	N447K	potential change in activity of BN8422_RS05080			1	1	1	
1078199	G	A	snp	intergenic region	-	-	NA	NA	potential change in transcription of BN8422_RS05445			1	1	1	
1087338	T	A	snp	pyruvate carboxylase	BN8422_RS05490	p	T1818A	N606K	potential change in activity of BN8422_RS05490					1	
1133949	C	T	snp	BppU family phage baseplate upper protein	BN8422_RS05845	p	C858T	no	none		1				1
1133954	G	A	snp	BppU family phage baseplate upper protein	BN8422_RS05845	p	G863A	S288N	potential change in activity of BN8422_RS05845		1				1
1135441/4	AAAC	AGGC	mnp	XkdX family protein	BN8422_RS05855	p	A147G, A148G	T50A	potential change in activity of BN8422_RS05855		1				1
1135476	T	A	snp	intergenic region	-	-	NA	NA	potential change in transcription of BN8422_RS05860		1				1
1135494	A	C	snp	intergenic region	-	-	NA	NA	potential change in transcription of BN8422_RS05860		1				1
1135500	G	A	snp	intergenic region	-	-	NA	NA	potential change in transcription of BN8422_RS05860		1				1
1135506	T	C	snp	intergenic region	-	-	NA	NA	potential change in transcription of BN8422_RS05860		1				1
1135530	A	G	snp	DUF2951 domain-containing protein	BN8422_RS05860	p	A21G	no	none		1				1
1135899	G	A	snp	intergenic region	-	-	NA	NA	potential change in transcription of BN8422_RS05865		1				1
1135960	T	C	snp	glucosaminidase domain-containing protein	BN8422_RS05865	p	T15C	no	none		1				1
1135964/9	AAAACA	AGACTA	mnp	glucosaminidase domain-containing protein	BN8422_RS05865	p	A19G, A21C, C22T	N8T	potential change in activity of BN8422_RS05865		1				1
1135975	A	G	snp	glucosaminidase domain-containing protein	BN8422_RS05865	p	A30G	no	none		1				1
1139592/7	TAGTAG	T**G**GT**G**G	mnp	glucosaminidase domain-containing protein	BN8422_RS05865	p	A48G, A51G	no	none		1				1
1198923	C		snp	cell division protein FtsQ/DivIB	BN8422_RS06205	p	C233G	S78C	potential change in activity of BN8422_RS06205				1		
1205715	A	T	snp	RNA-binding protein	BN8422_RS06240	p	A268T	T90S	potential change in activity of BN8422_RS06240			1	1	1	
1205724	G	C	snp	RNA-binding protein	BN8422_RS06240	p	G277C	E93Q	potential change in activity of BN8422_RS06240			1	1	1	
1206014/9	AGAAAT	ATATTT	mnp	RNA-binding protein	BN8422_RS06240	p	G568T, A570T, A571T	E190Y, M191L	potential change in activity of BN8422_RS06240			1	1	1	
1206047/56	ACAACTAATC	AGGGGGGGGC	mnp	RNA-binding protein	BN8422_RS06240	p	C601G, A602G, A603G, C604G,T605G, A606G, A607G,T608G	Q201G, L202G, I203G	potential change in activity of BN8422_RS06240			1	1	1	
1281987	A	C	snp	translation elongation factor Ts	tsf	p	A260C	E87A	potential change in activity of EF-Ts			1	1	1	
1314331/7	TTGTACT	TGTACTT	mmp	CDP-diacylglycerol--glycerol-3-phosphate 3-phosphatidyltransferase	pgsA	p	T273G, G274T, T275A, A276C, C277T	I91M, V92Y, L93F	potential change in activity of PgsA			1	1	1	
1316960	T	C	snp	recombinase RecA	recA	p	T780C	no	none			1	1	1	
1339788/93	TTGGGT	TGGGTT	mmp	GTPase HflX	hflX	p	T149G, G152T	L50W, G51V	potential change in activity of HflX			1	1	1	
1375467/72	CTTCTT	CCTTCT	mnp	transcriptional repressor LexA	lexA	m	A169G, G170A, A172G	R57E, R58G	potential change in activity of LexA			1	1	1	
1375505	A	C	snp	transcriptional repressor LexA	lexA	m	T135G	no	none			1	1	1	
1375516/9	TTGA	TGAA	mnp	transcriptional repressor LexA	lexA	m	C122T, A123C	S41F	potential change in activity of LexA			1	1	1	
1377825	T	A	snp	transketolase Tkt	tkt	p	T1315A	Y439L	potential change in activity of Tkt			1	1	1	
1377872/6	AACGT	ACGTT	mnp	transketolase Tkt	tkt	p	A1363C, C1364G, G1365T	T455T	potential change in activity of Tkt			1	1	1	
1379332	T	C	snp	CcdC family protein	BN8422_RS07100	p	T135C	no	none			1	1	1	
1379394/7	TAAT	TTGT	mnp	CcdC family protein	BN8422_RS07100	p	A198T, A199G	I67V	potential change in activity of BN8422_RS07100			1	1	1	
1379418	T	A	snp	CcdC family protein	BN8422_RS07100	p	T221A	V74D	potential change in activity of BN8422_RS07100			1	1	1	
1379468	A	G	snp	CcdC family protein	BN8422_RS07100	p	A271G	K91E	potential change in activity of BN8422_RS07100			1	1	1	
1379478	A	T	snp	CcdC family protein	BN8422_RS07100	p	A281T	K94I	potential change in activity of BN8422_RS07100			1	1	1	
1379482	A	C	snp	CcdC family protein	BN8422_RS07100	p	A285C	no	none			1	1	1	
1440347	T	G	snp	4-hydroxy-tetrahydrodipicolinate reductase	dapB	p	T419G	V140G	potential change in activity of DapB					1	
1441315/20	CTGCAA	CCTGCA	mnp	2,3,4,5-tetrahydropyridine-2,6-dicarboxylate N-acetyltransferase	dapD	p	T639C, G640T, C641G, A642C	A214C	potential change in activity of DapD			1	1	1	
1441330/4	AGCAA	AAGCA	mnp	2,3,4,5-tetrahydropyridine-2,6-dicarboxylate N-acetyltransferase	dapD	p	G654A, C655G, A666C	Q219A	potential change in activity of DapD			1	1	1	
1538870	A	G	snp	DUF1405 domain-containing protein	BN8422_RS07735	m	T160C	S54P	potential change in activity of BN8422_RS07735					1	1
1578805	G	C	snp	pyrroline-5-carboxylate reductase	proC	p	G611C	R204P	potential change in activity of ProC			1	1	1	
1578839/43	ACGTT	AACGT	mnp	pyrroline-5-carboxylate reductase	proC	p	C646A, G647C, T648G	R216T	potential change in activity of ProC			1	1	1	
1578848	T	A	snp	pyrroline-5-carboxylate reductase	proC	p	T654A	D218E	potential change in activity of ProC			1	1	1	
1578867/905	A**G**AAAAAATATTACCTCTAAAGGTGGTACGACACAAGCT	AAAAAAAAAATACCTCTAAAGGGGGTACAAAACAAGCCT	mmp	pyrroline-5-carboxylate reductase	proC	p	G673-, T681A, T683A, T696G, G702A, C704A, -711C	R225K, N227K, T279P, S280L, G283V, T284Q, T285N, Q286K, A287P	potential change in activity of ProC			1	1	1	
1579013	A	A**A**	ins	intergenic region	-	-	NA	NA	potential change in transcription of proC			1	1	1	
1655397	A	C	snp	translation elongation factor 4	lepA	m	T1164G	no	none			1	1	1	
1736464	C	T	snp	trigger factor	tig	m	G925A	V309I	potential change in activity of Tig			1	1	1	
1761009	T	T**T**	ins	intergenic region	-	-	NA	NA	potential change in transcription of BN8422_RS08930			1	1	1	
1851770	T	C	snp	intergenic region	-	-	NA	NA	potential change in transcription of BN8422_RS09300			1	1	1	
1861260/4	ATTCA	AATAA	mnp	intergenic region	-	-	NA	NA	potential change in transcription of BN8422_RS09355						1
1861277	A	T	snp	intergenic region	-	-	NA	NA	potential change in transcription of BN8422_RS09355						1

1861291	G	A	snp	intergenic region	-	-	NA	NA	potential change in transcription of BN8422_RS09355						1
1861301/7	TATGTT-A	TTTATT**CA**	mnp	intergenic region	-	-	NA	NA	potential change in transcription of BN8422_RS09355						1
1861309/13	GACGT	GTCTT	mnp	metalloregulator ArsR/SmtB family transcription factor	BN8422_RS09355	p	A6T, G8T	T2S	potential change in activity of BN8422_RS09355						1
1861325	G	T	snp	metalloregulator ArsR/SmtB family transcription factor	BN8422_RS09355	p	G19T	A7S	potential change in activity of BN8422_RS09355						1
1861330/4	ATTTT	AATAT	mnp	metalloregulator ArsR/SmtB family transcription factor	BN8422_RS09355	p	T25A, T27A	F9I	potential change in activity of BN8422_RS09355						1
1861340	G	A	snp	metalloregulator ArsR/SmtB family transcription factor	BN8422_RS09355	p	G34A	V12I	potential change in activity of BN8422_RS09355						1
1861356/61	GCAGAT	GTAGGT	mnp	metalloregulator ArsR/SmtB family transcription factor	BN8422_RS09355	p	C51T, A54G	no	none						1
1861370	C	T	snp	metalloregulator ArsR/SmtB family transcription factor	BN8422_RS09355	p	C64T	no	none						1
1861390	A	T	snp	metalloregulator ArsR/SmtB family transcription factor	BN8422_RS09355	p	A84T	no	none						1
1861394	T	C	snp	metalloregulator ArsR/SmtB family transcription factor	BN8422_RS09355	p	T88C	no	none						1
1861407/12	ATTTGT	ACTTAT	mnp	metalloregulator ArsR/SmtB family transcription factor	BN8422_RS09355	p	T102C, G105A	no	none						1
1861416	C	A	snp	metalloregulator ArsR/SmtB family transcription factor	BN8422_RS09355	p	C110A	A37E	potential change in activity of BN8422_RS09355						1
1861420	T	C	snp	metalloregulator ArsR/SmtB family transcription factor	BN8422_RS09355	p	T114C	no	none						1
1861432	T	A	snp	metalloregulator ArsR/SmtB family transcription factor	BN8422_RS09355	p	T126A	no	none						1
1861443/9	TTAGCTA	TAAGTCA	mnp	metalloregulator ArsR/SmtB family transcription factor	BN8422_RS09355	p	T138A, C140T, T141C	Y48H	potential change in activity of BN8422_RS09355						1
1861458/61	AAGC	AGTC	mnp	metalloregulator ArsR/SmtB family transcription factor	BN8422_RS09355	p	A153G, G154T	A52S	potential change in activity of BN8422_RS09355						1
1861753	T	C	snp	arsenite efflux transporter membrane subunit ArsB	arsB	p	T133C	F45S	potential change in activity of ArsB						1
1861758	C	T	snp	arsenite efflux transporter membrane subunit ArsB	arsB	p	C138T	no	none						1
1861763/8	TTTTCG	TATTAG	mnp	arsenite efflux transporter membrane subunit ArsB	arsB	p	T144A, C147A	F49L	potential change in activity of ArsB						1
1861794	T	A	snp	arsenite efflux transporter membrane subunit ArsB	arsB	p	T174A	no	none						1
1861800	G	A	snp	arsenite efflux transporter membrane subunit ArsB	arsB	p	G180A	no	none						1
1861808/16	TCTCAGTCA	TGGCTGTTA	mnp	arsenite efflux transporter membrane subunit ArsB	arsB	p	C189G, T190G, A192T, C195T	S64A	potential change in activity of ArsB						1
1861839/44	TAAAGT	TGAAAT	mnp	arsenite efflux transporter membrane subunit ArsB	arsB	p	A220G, G223A	K74E, V75I	potential change in activity of ArsB						1
1861940/5	TTGCTG	TAGCAG	mnp	arsenite efflux transporter membrane subunit ArsB	arsB	p	T321A, T324A	no	none						1
1861965	C	T	snp	arsenite efflux transporter membrane subunit ArsB	arsB	p	C345T	no	none						1
1861971	G	T	snp	arsenite efflux transporter membrane subunit ArsB	arsB	p	G351T	no	none						1
1862040	T	C	snp	arsenite efflux transporter membrane subunit ArsB	arsB	p	T420C	no	none						1
1862052	G	C	snp	arsenite efflux transporter membrane subunit ArsB	arsB	p	G432C	no	none						1
1862064	A	T	snp	arsenite efflux transporter membrane subunit ArsB	arsB	p	A444T	no	none						1
1862220	T	C	snp	arsenite efflux transporter membrane subunit ArsB	arsB	p	T600C	no	none						1
1862226/30	AGCGA	ATCCA	mnp	arsenite efflux transporter membrane subunit ArsB	arsB	p	G607T, G609C	A203S	potential change in activity of ArsB						1
1862706/11	CCTAAT	CATGGT	mnp	arsenite efflux transporter membrane subunit ArsB	arsB	p	C1087A, A1089G, A1090G	L363M, I364V	potential change in activity of ArsB						1
1862723/8	TTATCG	TCATAG	mnp	arsenite efflux transporter membrane subunit ArsB	arsB	p	T1104C, C1107A	no	none						1
186274/6	GTCCAA	GACCTA	mnp	arsenite efflux transporter membrane subunit ArsB	arsB	p	T1122A, A1125T	no	none						1
1862753/64	CACCGATAGGCT	CGCCAATTGGTT	mnp	arsenite efflux transporter membrane subunit ArsB	arsB	p	A1134G, G1137A, A1240T, C1143T	no	none						1
1862772	T	A	snp	arsenite efflux transporter membrane subunit ArsB	arsB	p	T1152A	no	none						1
1862779	C	T	snp	arsenite efflux transporter membrane subunit ArsB	arsB	p	C1159T	no	none						1
1862785	T	C	snp	arsenite efflux transporter membrane subunit ArsB	arsB	p	T1165C	no	none						1
1862790	C	T	snp	arsenite efflux transporter membrane subunit ArsB	arsB	p	C1170T	no	none						1
1862807	A	G	snp	arsenite efflux transporter membrane subunit ArsB	arsB	p	A1187G	D396G	potential change in activity of ArsB						1
1862811	T	G	snp	arsenite efflux transporter membrane subunit ArsB	arsB	p	T1191G	no	none						1
1862820	T	G	snp	arsenite efflux transporter membrane subunit ArsB	arsB	p	T1200G	no	none						1
1862826	C	A	snp	arsenite efflux transporter membrane subunit ArsB	arsB	p	C1206A	no	none						1
1862843/8	GTATCA	GAATTA	mnp	arsenite efflux transporter membrane subunit ArsB	arsB	p	T1224A, C1227T	no	none						1
1862852/7	TTACAA	TCACTA	mnp	arsenite efflux transporter membrane subunit ArsB	arsB	p	T1233C, A1236T	no	none						1
1862865	A	C	snp	arsenite efflux transporter membrane subunit ArsB	arsB	p	A1245C	no	none						1
1862872	A	G	snp	arsenite efflux transporter membrane subunit ArsB	arsB	p	A1252G	I418V	potential change in activity of ArsB						1
1862877	C	A	snp	arsenite efflux transporter membrane subunit ArsB	arsB	p	C1257A	no	none						1
1862881	A	T	snp	arsenite efflux transporter membrane subunit ArsB	arsB	p	A1261T	I421L	potential change in activity of ArsB						1
1862885/96	GGTTGTATCTAA	GTTTATACCTTA	mnp	arsenite efflux transporter membrane subunit ArsB	arsB	p	G1266T, G1269A, T1272C, A1275T	no	none						1
1862901	T	A	snp	arsenite efflux transporter membrane subunit ArsB	arsB	p	T1281A	no	none						1
1862939	A	G	snp	arsenate reductase (thioredoxin)	arsC	p	A9G	no	none						1
1862966	C	A	snp	arsenate reductase (thioredoxin)	arsC	p	C36A	no	none						1
1862972	A	T	snp	arsenate reductase (thioredoxin)	arsC	p	A42T	no	none						1
1862977/82	GAAGTC	GTAGCC	mnp	arsenate reductase (thioredoxin)	arsC	p	A48T, T51C	no	none						1
1863000/7	GCTAAACA	GGAAAGGA	mnp	arsenate reductase (thioredoxin)	arsC	p	C71G, T72A, A75G, C76G	A24G, Q26E	potential change in activity of ArsC						1
1863010/23	TCTTAGCGGATGAT	TATTGGGTGAAGGT	mnp	arsenate reductase (thioredoxin)	arsC	p	C81A, T84G, C86G, G87T, T90A, A92G	A29G, D30E, D31G	potential change in activity of ArsC						1
1863032	A	C	snp	arsenate reductase (thioredoxin)	arsC	p	A102C	no	none						1
1863047	C	T	snp	arsenate reductase (thioredoxin)	arsC	p	C117T	no	none						1
1863056	C	T	snp	arsenate reductase (thioredoxin)	arsC	p	C126T	no	none						1
1863068	C	T	snp	arsenate reductase (thioredoxin)	arsC	p	C138T	no	none						1
1863074	G	A	snp	arsenate reductase (thioredoxin)	arsC	p	G144A	no	none						1
1863096/9	GGCA	GATA	mnp	arsenate reductase (thioredoxin)	arsC	p	G167A, C168T	G56D	potential change in activity of ArsC						1
1863113	T	C	snp	arsenate reductase (thioredoxin)	arsC	p	T183C	no	none						1

1863119	A	G	snp	arsenate reductase (thioredoxin)	arsC	p	A189G	no	none						1
1863124/32	ATTTAATCG	ACTTGATTG	mnp	arsenate reductase (thioredoxin)	arsC	p	T195C, A198G, C201T	no	none						1
1863138	A	G	snp	arsenate reductase (thioredoxin)	arsC	p	A208G	N70D	potential change in activity of ArsC						1
1863188/92	CGTAA	CAATA	mnp	arsenate reductase (thioredoxin)	arsC	p	G259A, T260A, A261T	V87N	potential change in activity of ArsC						1
1863197	C	T	snp	arsenate reductase (thioredoxin)	arsC	p	C267T	no	none						1
1863200/3	TTCT	TATT	mnp	arsenate reductase (thioredoxin)	arsC	p	T271A, C272T	S91I	potential change in activity of ArsC						1
1863210	A	C	snp	arsenate reductase (thioredoxin)	arsC	p	A280C	T94P	potential change in activity of ArsC						1
1863215	T	C	snp	arsenate reductase (thioredoxin)	arsC	p	T285C	no	none						1
1863221	G	A	snp	arsenate reductase (thioredoxin)	arsC	p	G291A	no	none						1
1863227	A	G	snp	arsenate reductase (thioredoxin)	arsC	p	A297G	no	none						1
1863236	A	T	snp	arsenate reductase (thioredoxin)	arsC	p	A306T	no	none						1
1863248	T	A	snp	arsenate reductase (thioredoxin)	arsC	p	T318A	no	none						1
1889575/60	TTGCCG	TCACAG	mnp	intergenic region	-	-	NA	NA	potential change in transcription of PepA1						1
1889564	A	G	snp	intergenic region	-	-	NA	NA	potential change in transcription of PepA1						1
1889572	G	A	snp	intergenic region	-	-	NA	NA	potential change in transcription of PepA1						1
1889619	A	A**A**	ins	intergenic region	-	-	NA	NA	potential change in transcription of PepA1						1
1889781	C	G	snp	intergenic region	-	-	NA	NA	potential change in transcriptional termination of PepA1						1
1889785/6	A**A**	A	del	intergenic region	-	-	NA	NA	potential change in transcriptional termination of PepA1						1
1911271	G	A	snp	lanthionine synthetase C family protein	BN8422_RS09640	m	G272A	A91V	potential change in activity of BN8422_RS09640		1				
1923290	C	T	snp	protoporphyrinogen oxidase	hemY	m	G1363A	G455S	potential change in activity of HemY			1	1	1	
1923314/8	CAACC	CCAAC	mmp	protoporphyrinogen oxidase	hemY	m	G1336T, T1338G	V446G	potential change in activity of HemY			1	1	1	
1996699	T	G	snp	hypothetical protein	BN8422_RS10190	m	A600C	no	none			1	1	1	
1972907	G	C	snp	intergenic region	-	-	NA	NA	potential change in transcription of BN8422_RS10085						1
1983554	T	A	snp	BppU family phage baseplate upper protein	BN8422_RS10145	m	T718A	N240I	potential change in transcription of BN8422_RS10145						1
1983903	G	A	snp	BppU family phage baseplate upper protein	BN8422_RS10145	m	G369A	no	none		1				1
1986622	A	G	snp	intergenic region	-	-	NA	NA	potential change in transcription of BN8422_RS10155		1				1
1986628	C	T	snp	intergenic region	-	-	NA	NA	potential change in transcription of BN8422_RS10155		1				1
1986634	T	G	snp	intergenic region	-	-	NA	NA	potential change in transcription of BN8422_RS10155		1				1
1986652	A	T	snp	intergenic region	-	-	NA	NA	potential change in transcription of BN8422_RS10155		1				
2007942	A	T	snp	DUF1381 domain-containing protein	BN8422_RS10290	m	A17T	V6D	potential change in activity of BN8422_RS10290						1
2007951	T	G	snp	DUF1381 domain-containing protein	BN8422_RS10290	m	A8T	Q3H	potential change in activity of BN8422_RS10290		1				
2008002	A	C	snp	dUTP diphosphatase	BN8422_RS10295	m	A529C	Y177D	potential change in activity of BN8422_RS10295		1				
2008008	T	G	snp	dUTP diphosphatase	BN8422_RS10295		T523G	K175Q	potential change in activity of BN8422_RS10295		1				
2008310	A	C	snp	dUTP diphosphatase	BN8422_RS10295	m	A221C	F74C	potential change in activity of BN8422_RS10295						1
2018862/3	AA	ATCGAA	ins	helix-turn-helix transcriptional regulator	BN8422_RS10415	m	NA	NA	ORF not annotated in AP009351.1			1	1	1	
2020036/8	AA-C	AG**G**C	mnp	helix-turn-helix transcriptional regulator	BN8422_RS10415	m	NA	NA	ORF not annotated in AP009351.1			1	1	1	
2044040	T	A	snp	intergenic region	-	-	NA	NA	none					1	
2044055	G	C	snp	intergenic region	-	-	NA	NA	none					1	
2048395	A	G	snp	sodium/proline symporter PutP	putP	p	A224G	E75G	potential change in activity of PutP					1	1
2153627/8	AT	A-**IS1811**-T	ins	quorum-sensing response regulator AgrA	BN8422_RS11235	p	multiple	M25G, I26S, E27S, E28S, K29F P30M, M31V, E32G, I33R, A34*	Insertion of a frameshifted ISL3-like element IS1181 family transposase leading to a truncated AgrA						1
2153725	C	A	snp	LytTR family DNA-binding domain-containing protein AgrA	BN8422_RS11235	p	C168A	Y56*	truncated AgrA					1	
2159274	A	T	snp	ammonium transporter	BN8422_RS11260	m	A340T	S114T	potential change in activity of TsaB		1				
2168120	C	T	snp	tRNA (adenosine(37)-N6)-threonylcarbamoyltransferase complex dimerization subunit type 1 TsaB	tsaB	m	G375A	M125I	potential change in activity of BN8422_RS11260					1	1
2367325/30	TATA**AT**	TTTA-T	mnp	30S ribosomal protein S5	rpsE	m	T491-, T493A	L164*, Y165-, N166-	C-terminal truncation of RpsE			1	1	1	
2432513	T	A	snp	intergenic region	-	-	NA	NA	potential change in transcription of fdhF			1	1	1	
2474077	T	C	snp	hypothetical protein	BN8422_RS12975	p	T279C	no	none					1	1
2505074/7	AATA	ATAA	mnp	intergenic region	-	-	NA	NA	none			1	1	1	
2505101/2	A**A**	A	del	intergenic region	-	-	NA	NA	none			1	1	1	
2505126/7	T**T**	T	del	intergenic region	-	-	NA	NA	none			1	1	1	
2505141	T	G	snp	intergenic region	-	-	NA	NA	none			1	1	1	
2505150/1	A**A**	A	del	intergenic region	-	-	NA	NA	none			1	1	1	
2510394	C	A	snp	cation:dicarboxylase symporter family transporter	BN8422_RS13155	m	G764T	G255V	potential change in activity of BN8422_RS13155			1	1	1	
2533055/6	T**T**	T	del	intergenic region	-	-	NA	NA	potential change in transcription of BN8422_RS13260			1	1	1	
2533168	T	A	snp	zinc ABC transporter substrate-binding lipoprotein AdcA	adcA	m	A1551T	*517Y	C-terminal elongation of AdcA in AP009351.1			1	1	1	
2533196	T	G	snp	zinc ABC transporter substrate-binding lipoprotein AdcA	adcA	m	A1523C	Q508P	potential change in activity of AdcA			1	1	1	
2533236	T	A	snp	zinc ABC transporter substrate-binding lipoprotein AdcA	adcA	m	A1483C	N495Y	potential change in activity of AdcA			1	1	1	
2533243/6	TTCT	TCTT	mnp	zinc ABC transporter substrate-binding lipoprotein AdcA	adcA	m	G1456A, A1457G	E492R	potential change in activity of AdcA			1	1	1	
2533314	T	G	snp	zinc ABC transporter substrate-binding lipoprotein AdcA	adcA	m	A1405C	N469H	potential change in activity of AdcA			1	1	1	
2535540/4	CTCC	CCTC	mnp	intergenic region	-	-	NA	NA	potential change in transcription of BN8422_RS13275			1	1	1	

2542059/61	GTAA	GGTA	mnp	multidrug efflux MFS transporter MdeA	mdeA	m	T92A, A93C	L31Y	potential change in activity of MdeA			1	1	1	
2542082/101	TCATT**T**AATAATCCAAAA**A**A	TTTCATTAATAATCCAAAAA	mmp	multidrug efflux MFS transporter MdeA	mdeA	m	T53-, A66-, -70A, -71A	NA	N-termially truncated mdeA ORF in AP009351.1			1	1	1	
2570240	T	G	snp	sodium:proton antiporter	BN8422_RS13470	p	T1583G	V528G	potential change in activity of BN8422_RS13470			1	1	1	
2570252/9	AGACACAC	AAACCCCC	mnp	sodium:proton antiporter	BN8422_RS13470	p	G1596A, A1599C, A1601C	H534P	potential change in activity of BN8422_RS13470			1	1	1	
2570276	T	G	snp	sodium:proton antiporter	BN8422_RS13470	p	T1619G	V540G	potential change in activity of BN8422_RS13470			1	1	1	
2570299	A	C	snp	sodium:proton antiporter	BN8422_RS13470	p	A1642G	K548Q	potential change in activity of BN8422_RS13470			1	1	1	
2570315	G	A	snp	sodium:proton antiporter	BN8422_RS13470	p	G1658A	R553K	potential change in activity of BN8422_RS13470			1	1	1	
2679437	A	C	snp	glucose-specific PTS transporter subunit IIBC	ptsG	m	A947C	V316G	potential change in activity of PtsG						1
2685937	T	C	snp	intergenic region	-	-	NA	NA	none						1
2686018/23	TATTAT	TTTTCT	mnp	intergenic region	-	-	NA	NA	none						1
2686028	A	G	snp	intergenic region	-	-	NA	NA	none						1
2686032/39	TTCGCTA	TCCACCA	mnp	intergenic region	-	-	NA	NA	none						1
2686062	T	C	snp	intergenic region	-	-	NA	NA	none						1
2686070	G	T	snp	intergenic region	-	-	NA	NA	none						1
2744980	G	G**G**	ins	intergenic region	-	-	NA	NA	potential change in transcription of BN8422_RS14350			1	1	1	

Bold	Additional nucleotide(s) present in REF or ALT
	Gene alterations found in Newman (AP009351.1), NYU Newman (CP023390.1) and Newman D2C NY (CP023391.1)
	Gene alterations found in Newman HOM (CP160003.1) only
	Gene alterations found in Newman (AP009351.1) only
	Gene alterations found in NYU Newman (CP023390.1) only
	Gene alterations found in Newman D2C HOM (CP160002.1) only
	Gene alterations found in Newman D2C NY (CP023391.1) only
	Gene alterations found in Newman D2C NY (CP023391.1) and Newman D2C HOM (CP160002.1) only
	Gene alterations found in Newman HOM (CP160003.1) and Newman D2C HOM (CP160002.1) only
	Gene alterations found in Newman (AP009351.1) and NYU Newman (CP023390.1) only
